# Rare germline variants contribute to glioma predisposition: Whole-genome analysis of a regional cohort of glioma patients

**DOI:** 10.1093/noajnl/vdag038

**Published:** 2026-02-12

**Authors:** Adam Rosenbaum, Carl Wibom, Austin Hammermeister Suger, Raphaela Pensch, Ananya Roy, Thomas Brännström, Matilda Rentoft, Karin Forsberg-Nilsson, Kerstin Lindblad-Toh, Sara Lindström, Anna Margareta Dahlin, Beatrice Melin

**Affiliations:** Department of Diagnostics and Intervention, Oncology, Umeå University, Umeå, Sweden; Department of Diagnostics and Intervention, Oncology, Umeå University, Umeå, Sweden; Department of Epidemiology, University of Washington, Seattle; Department of Medical Biochemistry and Microbiology, Uppsala University, Uppsala, Sweden; Science for Life Laboratory, Uppsala, Sweden; Science for Life Laboratory, Uppsala, Sweden; Department of Immunology, Genetics and Pathology, Uppsala University, Uppsala, Sweden; Department of Medical Biosciences, Umeå University, Umeå, Sweden; Department of Diagnostics and Intervention, Oncology, Umeå University, Umeå, Sweden; Science for Life Laboratory, Uppsala, Sweden; Department of Immunology, Genetics and Pathology, Uppsala University, Uppsala, Sweden; Department of Medical Biochemistry and Microbiology, Uppsala University, Uppsala, Sweden; Science for Life Laboratory, Uppsala, Sweden; Broad Institute of MIT and Harvard, Cambridge; Department of Epidemiology, University of Washington, Seattle; Division of Public Health Sciences, Fred Hutchinson Cancer Center, Seattle; Department of Diagnostics and Intervention, Oncology, Umeå University, Umeå, Sweden; Department of Diagnostics and Intervention, Oncology, Umeå University, Umeå, Sweden

**Keywords:** adult glioma, glioma predisposition, rare variants, whole-genome sequencing

## Abstract

**Background:**

Gliomas are the most common malignant primary tumor of the central nervous system and show a high mortality, particularly at higher grades. Cancer predisposition syndromes and common low-penetrance single nucleotide polymorphisms have been shown to contribute to glioma risk, but the contribution of rare germline variants remains incompletely understood. Here, we investigated rare germline variants in glioma patients.

**Methods:**

We performed whole-genome sequencing on 113 glioma patients from Northern Sweden, analyzing rare germline variants across 651 genes. Variants were compared to population controls (ACpop, gnomAD) and validated in TCGA glioma data, a UK Biobank glioma nested case–control study, and a separate cohort of 105 Swedish glioblastomas.

**Results:**

17.6% of glioma cases carried a Pathogenic or Likely Pathogenic (P/LP) variant within 1 of the 651 genes, and the number of alleles carrying a P/LP was significantly more than in the reference data (*P* = $3.2\times 10^{-3}$). Many of the observed candidate genes also harbored P/LP variants in our Swedish validation cohort. Overall, gene-based comparison of rare coding variants indicated an enrichment in several genes, including *TP53*, *CREBBP*, and *DNMT3A*.

**Conclusions:**

Rare P/LP germline variants were more frequent among glioma patients than in the reference population within our predefined gene set. These results suggest a contribution of rare germline variants to glioma risk, particularly in genes involved in DNA repair. While several genes are indicated as enriched with rare variants, only *TP53* validates across all 3 patient sets.

Key Points

- Rare pathogenic germline variants in patients with glioma are common.
- Variant Gene burden analysis highlights that rare variants are more common in *TP53.*

Importance of the Study

In this study, we analyzed whole-genome germline data from a regional cohort of glioma patients in Northern Sweden without a known family history of glioma. A notable proportion of patients carried rare P/LP germline variants, of which many resided in DNA repair genes. Gene variant burden analysis further showed several genes with rare coding variants common in glioma patients as compared to the healthy population. These results suggest that germline genetics play a broader role in glioma susceptibility than previously thought. This work highlights the prospect of integrating germline genetics in risk stratification and the possibility for individualized targeted therapy to be tested in the future.

Gliomas are the most common malignant central nervous system (CNS) tumors in adults.^1^ Low-grade gliomas (LGGs) are often treated with favorable patient survival. However, glioblastomas (GBMs), a WHO grade 4 glioma, have a median survival of only 12-14 months after available treatment regimens.^2–4^ The age at diagnosis for GBMs most often occurs at 56-72 years of age, while other subtypes, such as oligodendroglioma and astrocytomas of lower grades, typically onset earlier.^1^ Cancers arising at younger ages are more often associated with underlying germline variants.^5^^,^^6^

Little is known about the etiology of glioma, and the only established environmental risk factor is exposure to ionizing radiation.^7^ Moreover, the occurrence of glioma is inversely associated with respiratory allergies,^8^ suggesting that an immunological response is protective. Having a first-degree relative with glioma is associated with a 2-fold increase in risk,^9^^,^^10^ and inherited variants associated with rare genetic cancer-predisposition disorders, such as Li-Fraumeni, neurofibromatosis, and Turcot’s syndrome, increase the risk of glioma.^11^ Variants in mismatch repair genes (*MLH1*, *MSH2*, *MSH6*, and *PMS2*) leading to Lynch syndrome have also been linked to glioma predisposition, particularly when both gene copies carry a variant resulting in biallelic mismatch repair deficiency.^12^^,^^13^ During the past 2 decades, genome-wide association studies (GWAS) have identified 25 common single nucleotide polymorphisms (SNPs) associated with glioma risk.^14–19^ Family based studies have further identified rare variants in *POT1*,^20^  *POLE*, and *POLD1*.^21^ These were further confirmed in a whole-genome sequencing (WGS) effort of 304 families, where a broad spectrum of rare variants was detected in genes related to cancer development, including *POLE*, *POLD1*, *PMS2*, and *MLH* and additionally found novel associations to glioma for genes *HERC2*, *MYO7A*, *IP6K1*, *ADAMTS8*, and *LRRK2.*^22^ Few variants were shared between families in this previous study. We propose that rare germline variants may also play a role in development of sporadic gliomas. Our study hence focuses on gliomas lacking a known familial component.

This study aims to investigate if rare deleterious germline variants contribute to glioma development in a regional Swedish population. We conducted WGS in 113 glioma patients and annotated variants in 651 candidate genes based on 3 groups: (1) known cancer-predisposition genes, (2) genes related to glioma etiology, and (3) genes frequently somatically mutated in cancer. All subsequent analyses were restricted to this predefined gene set.

## Methods

### Samples

Blood and tumor samples from glioma patients have been collected at Umeå University Hospital since 2005 and within the U-CAN project since 2010.^23^ U-CAN is a Swedish multi-center cancer initiative with participating sites including Umeå in Northern Sweden and Uppsala. The Northern Sweden cases (Umeå site) described here were used as the discovery dataset in this study; independent Swedish cases from the Uppsala site are described under the validation datasets. All participants provided their informed consent to be part of the U-CAN project, and we obtained ethical approval from the regional ethical review board in Umeå (2011-308-31M, 2018-209-32M) for this study. We included 2 sets of glioma patients.

1. Early onset (*n* = 46): Glioma cases diagnosed between the ages 18 and 40.
2. Tumor-Germline (*n* = 73): Glioma cases with sufficient tumor volume for sequencing. Six of these were also part of the early onset individuals.

*IDH*-status was available for all Tumor-Germline cases. For the early onset cases, *IDH1*/*2* status was unknown for 16 of 46 cases and was labeled as “unknown,” and should not be interpreted as *IDH*-wildtype.

In glioma cases where tumor tissue was available, tumors were classified according to the WHO 2016 guidelines for CNS tumors. The WHO 2016 classifications were harmonized to the WHO 2021 recommendations where possible. As an example, 3 tumors classified as *IDH*-mutated GBMs according to WHO 2016 were reclassified as astrocytoma of grade 4 according to the WHO 2021 recommendations. Where *IDH* status was unavailable, WHO 2021 entities requiring *IDH* status were not inferred. Furthermore, more unusual glioma entities with less than 3 individuals were classified as “others” to ensure anonymity of our study participants.

As a regional reference to our patients from Northern Sweden, we used ACpop,^24^ a population database with germline allele frequencies based on 300 individuals from Västerbotten County, all without a current or previous cancer diagnosis at the age of 80. Because ACpop variants were originally aligned to hg19, we applied Picard LiftOver to convert the coordinates to GRCh38 prior to analysis. These data were used as a reference population for our Northern Swedish cases.

### Validation Datasets

To validate any results, we used the following external datasets.

1. TCGA Glioma Dataset (*n* = 806): We used germline calls from TCGA of 10 380 individuals as described by Huang et al.^25^ We downloaded the compressed vcf file (PCA.r1.TCGAbarcode.merge.tnSwapCorrected.10389.vcf.gz) from the Genomic Data Commons using controlled access and the gdc data transfer tool. We subset the data to 806 European individuals having either LGG or high-grade glioma. Three hundred thirty-five of these cases were denoted as GBM, and 471 were denoted as LGG. Out of the 471 LGG cases, 396 had a known mutation in *IDH1*/*2*. Data were lifted over from hg19 to GRCh38 using Picard LiftOver.
2. gnomAD v2.1.1 (Exon, European non-Finnish non-cancer subset): We used gnomAD as a control population for the glioma cases in TCGA.
3. UK Biobank (UKB; *n* = 9148: 833 cases and 8315 controls):^26^ WES data from European ancestry individuals diagnosed with glioma and a subset of those not diagnosed with any cancer. Analyses were conducted on the UKB Research Analysis Platform under Application # 95770.
4. Uppsala GBM cases (*n* = 105) from U-CAN: Germline variant calls were derived from WGS of blood samples from GBM patients in Uppsala, Sweden. Of these, 6 were found to have an *IDH1*/*2* after sequencing of the tumors. These were considered as grade 4 astrocytomas.

**Figure 1.**
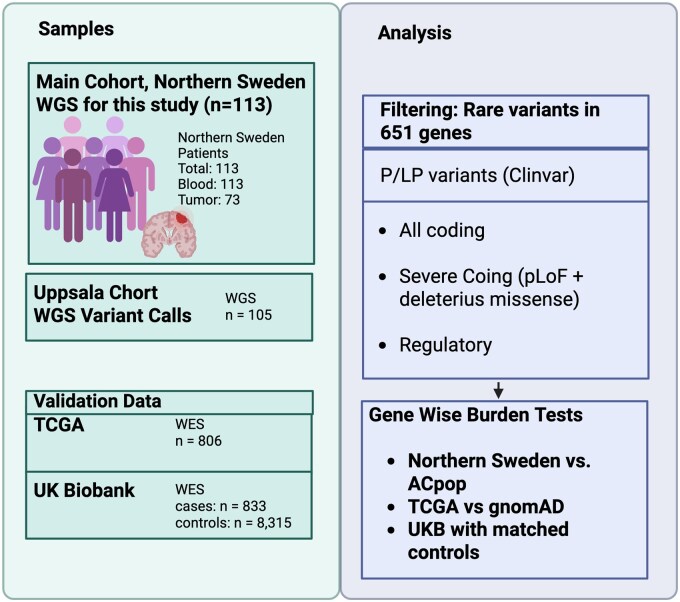
Overview of study design and analytical workflow. Schematic summary of the study cohorts and analysis steps. The main cohort (*n* = 113, Northern Sweden) underwent whole-genome sequencing and was analyzed together with validation cohorts from Uppsala (*n* = 105), TCGA (*n* = 806), and UK Biobank (*n* = 833 cases, *n* = 8315 controls). Created in BioRender. https://BioRender.com/dt0q643. P/LP, Pathogenic or Likely Pathogenic; pLoF, predicted loss of function; UKB, UK Biobank; WGS, whole-genome sequencing.

An overview of all samples used in this study can be seen in Figure 1 and Table S2.

### Whole-Genome Sequencing

DNA from blood and tumor samples in the U-CAN consortium was sent for WGS at the SNP&SEQ Technology Platform in Uppsala. Sequencing libraries were prepared from 1 µg DNA using TruSeq PCRfree DNA sample preparation kit and unique dual indexes, targeting an insert size of 350 bp.

For the early onset glioma cases (*n* = 46), paired-end 150 bp sequencing was performed on the HiSeqX platform using v2.5 sequencing chemistry with a target coverage of 30×.

For matched tumor-normal cases (*n* = 73), paired-end 150 bp sequencing was performed on the NovaSeq 6000 platform using v.1.5 sequencing chemistry, with a targeted coverage of 30× for germline and 90× for tumors. The tumor tissue samples were previously annotated to have at least 50% tumor material. For the 6 individuals sequenced twice (included in both groups), the downstream germline analysis was done with the NovaSeq 6000 data.

For the Uppsala U-CAN samples, DNA was extracted from whole blood samples. All samples above the threshold value for library preparation were sequenced using the Illumina HiSeqX and True Seq PCR-free method. Minimum target depths of coverage of 30× was set for the samples. This yielded a paired-end 151-bp read length for each sample.

### Variant Calling

For the samples from Northern Sweden, FASTQ files were aligned to the reference sequence GRCh38 using bwa (v.0.7.17),^27^ and single-nucleotide variants (SNV) and small insertions/deletions (indel) calling was performed using GATK (v4.1.7.0) following the best practice workflow, using HaplotypeCaller followed by joint genotyping.^28^ Variants were filtered using Variant Quality Score Recalibration, and only variants marked as PASS were included in the analysis. Variants were annotated using Variant Effect Predictor (VEP), together with AlphaMissense v.1,^29^ zoonomia v.1,^30^ spliceAI v.1,^31^ and SweGen (variant allele frequencies for 1000 Swedish individuals).^32^

The Uppsala cohort’s sequencing data were preprocessed with the nf-core sarek pipeline (v2.3)^33^ using Nextflow (v19.04.1). In brief, the reads were mapped to the human reference genome GRCh37 with bwa (v0.7.17). Duplicates were marked, and base quality scores were recalibrated with GATK (v4.0.9.0). Variant calling was performed with HaplotypeCaller.

Structural variants (SVs) for all 113 northern Sweden glioma cases were called with Delly (v1.1.6)^34^ and Manta (v1.6.0).^35^ Only consensus SVs between Delly and Manta were retained. The SVs were further aggregated across all samples using SURVIVOR (v1.0.3).^36^ The SVs were annotated with VEP, and only variants with an impact of “HIGH” with regard to any of the target genes were considered. Somatic SVs were called using Manta with the paired tumor-normal BAM files as input.

For the 73 tumor-normal pairs, SNVs were called with GATK mutect2 and strelka2 (v2.8.4) with the blood and tumor BAM files as input. All autosomal coding regions were evaluated, and only consensus calls between mutect2 and strelka2 were considered. In addition somatic copy number alterations were called with CNVkit (v0.9.8).^37^

Tumor mutational burden (TMB) was calculated as the number of somatic SNVs per Mb across the autosomal coding regions used in the variant calling, using consensus variants from Mutect2 and Strelka2.

### Gene Set Selection

The genes investigated in this study were limited to genes that are related to known cancer susceptibility genes in Rahman 2014^38^ (*n* = 114), cosmic census v97 commonly mutated genes across all cancers^39^ (*n* = 579), genes within proximity of glioma GWAS SNPs^14^^,^^40^ (*n* = 39), genes related to brain tumor predisposition syndromes^41^ (*n* = 17), and genes implicated in a study with familial gliomas^22^ (*n* = 25). In total, this resulted in 651 unique genes (Table S1). As this gene set was biased toward genes involved in cancer, any results should not be interpreted as genome-wide enrichment of any gene category.

### Variant Filtering

**Figure 2.**
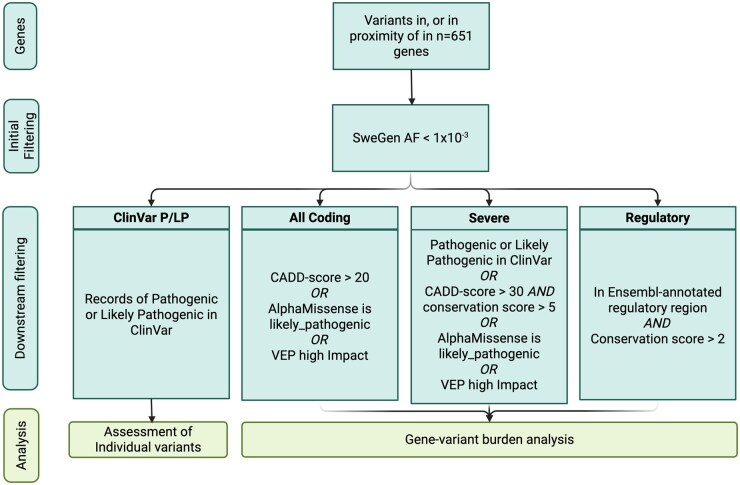
Overview of variant filtering and analysis strategy. Schematic outline of filtering steps and following analysis strategies. Created in BioRender. https://BioRender.com/e62k929.

SNVs/indels and SVs were initially filtered to include only those within ±100 kb of the 651 target genes. Additionally, variants with a SweGen allele frequency >1 × 10^−3^ were excluded. Variants were subsequently filtered according to 4 different filtering criteria (Figure 2): Coding variants, variants with records in ClinVar, severe coding variants, and regulatory variants. Pathogenic or Likely Pathogenic (P/LP) status was assigned only when a variant had records as such in ClinVar.

### Gene Variant Burden Test

For each gene, we compared the frequency of rare variants using the 3 above-mentioned filter strategies (all coding variants, severe coding variants, and regulatory variants). The allele frequencies in the 113 glioma patients were compared with the ACpop allele frequencies using Fisher’s exact 1-sided test, and the allele frequencies in the TCGA gliomas were compared with gnomAD allele frequencies. Gene-specific 1-sided Fisher’s exact test was performed to evaluate whether the glioma cases had a significantly higher frequency of rare variants compared to the controls. All genes with an unadjusted *P*-value below 0.1 from the Northern Sweden glioma patients, using any filtering strategy, were tested in TCGA and the UKB. We report unadjusted *P*-values.

### UKB-Matched Case–Control Study

We identified 833 European genetic ancestry individuals with WES data in the full UKB dataset who were diagnosed with brain tumor (ICD-10: C71 or ICD-9: 191) and had histology codes (ICD-O-3) indicating GBM (*N* = 613; codes: 9440-9442) or LGG (*N* = 220; codes: 9380-9383, 9400, 9401, 9410, 9411, 9450, 9451). We then identified European ancestry individuals without any cancer registry reported or self-reported cancer diagnoses to serve as potential controls. We removed potential controls who had estimated kinship coefficients above a threshold first degree or closer genetic relatedness (0.177) with a glioma case or other potential control. We then randomly selected eligible controls who were in the same age category ([37-40], [40-55], [55-65], or [65-87] years) as cases, with an approximate 1:10 case–control frequency matching, resulting in 8315 controls.

We conducted gene-based burden tests using REGENIE v3.3.^42^ The REGENIE models for glioma were adjusted for age, sex, and the first 10 genetic principal components. We included the first 10 PCs as fixed effects to control for broad-scale population structure within the UKB European-ancestry subset. The choice of 10 PCs was guided by inspection of PC plots for the included cases and controls and is consistent with prior UKB analyses indicating that ∼5-10 PCs capture the major axes of ancestry variation in this population.^43^ The REGENIE burden tests were conducted for variants with minor allele frequencies (MAF) less than 1% and Combined Annotation Dependent Depletion (CADD; v1.6) scores >20 in the 52 candidate genes.^44^ The gene-based tests in REGENIE used 3 variant sets for each gene based on the consequence predicted by Ensembl VEP (v122.0) for the variant.^45^ One group of gene-based sets included predicted loss of function (pLoF; VEP consequences: “splice_acceptor_variant,” “splice_donor_variant,” “stop_gained,” “stop_lost,” “frameshift_variant,” “start_loss”) variants with CADD scores >20. Another included pLoF variants and missense variants with CADD scores >20. The final included variants with CADD scores >20 and any predicted consequence. We also extracted MAF and minor allele counts for each tested variant separately for cases and controls.

## Results

### Samples

We collected a total of 113 consecutively recruited glioma patients from Northern Sweden through the U-CAN project (Table 1). Of these, 46 had their diagnosis between 18 and 40 years of age (21-39 years, median 32 years). Seventy-three individuals, ranging from age 30 to 79 (median 60 years) had both DNA from tumor tissue and blood sequenced. Glioblastoma was the most common diagnosis.

**Table 1.** Summary of sample characteristics

Characteristics	<40 years, *N* = 46	≥40 years, *N* = 67
**Diagnosis**		
** Astrocytoma (grade 2)**	9 (20%)	3 (4.5%)
** Astrocytoma (grade 3)**	3 (6.5%)	2 (3%)
** Astrocytoma (grade 4)**	3 (6.5%)	2 (3%)
** Glioblastoma (grade 4)**	14 (30%)	53 (79%)
** Gliosarcoma (grade 4)**	0 (0%)	3 (4.5%)
**Oligodendroglioma (grades 2-3)**	11 (24.3%)	2 (3%)
**Pilocytic astrocytoma (grade 1)**	3 (6.5%)	0 (0%)
** Others^a^**	3 (6.5%)	2 (3%)
**Sex**		
** Female**	22 (48%)	24 (36%)
** Male**	24 (52%)	43 (64%)
**IDH-status**		
** mut**	20 (43%)	7 (10%)
** wt**	10 (22%)	60 (90%)
** Unknown**	16 (35%)	0 (0%)
**Tumor WGS**	6 (13%)	67 (100%)

Description of 113 glioma cases from Northern Sweden included in this study

a Glioma entities with no more than 2 individuals are aggregated into this group.

Abbreviation: WGS, whole-genome sequencing.

### Pathogenic and Likely Pathogenic Variants in Glioma Patients

The set of all coding variants was examined in 113 individuals. Twenty of 113 individuals (17.6%) had a variant listed as P/LP in ClinVar. In contrast, we only found 22 variants that met the same criteria in ACpop. Comparing the number of alleles with P/LP between our patients and ACpop, we found that the occurrence of P/LP variants was significantly more common in the glioma patients (*P* = $3.2\times 10^{-3}$, Fisher’s exact test) (Table 2). We compared the number of P/LP in the early onset patients (<40 years) with the late-onset (≥40 years) and found no enrichment of P/LP variants in the younger patients (Fisher’s exact, *P* = .42).

**Table 2.** Pathogenic and likely pathogenic variants in glioma patients from Northern Sweden

Position	HGNCp	Clinvar entry	Gene	Consequence (VEP)	Diagnosis	IDH-status	Age at diagnosis <40
**chr1:43338634: G: C**	p.Arg102Pro	P	MPL	missense_variant	Glioblastoma (grade 4)	wt	No
**chr2:214781360: C: CT**	p.Asp172ArgfsTer10	P	BARD1	frameshift_variant	Glioblastoma (grade 4)	wt	No
**chr3:14148560: A: G**	.	LP	XPC	splice_donor_variant	Oligodendroglioma (grade 2)	mut	No
**chr3:48572138: G: A**	p.Arg2338Ter	P	COL7A1	stop_gained	Astrocytoma (grade 4)	mut	Yes
**chr5:224432: C: T**	p.Arg75Ter	P/LP	SDHA	stop_gained	Astrocytoma (grade 2)	mut	Yes
**chr7:142751919: C: T**	p.Arg116Cys	P/LP	PRSS1	missense_variant	Astrocytoma (grade 2)	unknown	Yes
**chr8:144513412: G: A**	p.Gln757Ter	P	RECQL4	stop_gained	Glioblastoma (grade 4)	wt	No
**chr8:144515891: C: T**	.	LP	RECQL4	splice_acceptor_variant	Astrocytoma (grade 2)	mut	Yes
**chr9:97675579: G: A**	p.Arg228Ter	P	XPA	stop_gained	Oligodendroglioma (grade 2)	mut	Yes
**chr10:70598772: C: T**	p.Gly317Arg	P	PRF1	missense_variant	Glioblastoma (grade 4)	unknown	Yes
**chr11:77156022: T: A**	p.Ile134Asn	LP	MYO7A	missense_variant	Astrocytoma (grade 3)	mut	No
**chr15:38351477: CAGAG: C**	p.Gly385IlefsTer20	P/LP	SPRED1	frameshift_variant	Pilocytic astrocytoma (grade 1)	wt	Yes
**chr16:89815966: T: A**	p.Lys34Ter	P	FANCA	stop_gained	Astrocytoma (grade 2)	mut	Yes
**chr17:7674972: C: T**	.	P	TP53	splice_acceptor_variant	Glioblastoma (grade 4)	wt	No
**chr17:7675139: C: T**	p.Arg158His	P/LP	TP53	missense_variant	Glioblastoma (grade 4)	unknown	Yes
**chr17:43093514: C: A**	p.Glu673Ter	P	BRCA1	stop_gained	Glioblastoma (grade 4)	wt	No
**chr17:58709926: GT: G**	p.Thr259LeufsTer4	P/LP	RAD51C	frameshift_variant	Glioblastoma (grade 4)	wt	No
**chr19:45352801: C: G**	p.Arg616Pro	P	ERCC2	missense_variant	Astrocytoma (grade 2)	mut	Yes
**chr22:20993977: G: A**	p.Trp469Ter	P	LZTR1	stop_gained	Glioblastoma (grade 4)	wt	No
**chr22:28725338: T: C**	p.Arg117Gly	P/LP	CHEK2	missense_variant	Glioblastoma (grade 4)	wt	No

Abbreviations: P/LP, pathogenic or likely pathogenic; VEP, variant effect predictor.

In addition to P/LP variants, we observed 40 predicted loss-of-function variants not present in ClinVar (Table S4), which may represent P/LP variants without a formal classification. Some of these variants were found in LoF-intolerant genes (pLI >0.9), including variants in *KMT2C*, *SPRED1*, *USP8*, *CREBBP*, *CDK12*, *SMAD4*, and *MALT1*.

Among the 20 ClinVar variants we observed, 12 were located in genes included in DNA repair or homologous recombination (*BRCA1*, *BARD*, *XPC*, *XPA*, *ERCC2*, *RECQL4* [2 variants], *FANCA*, *RAD51C*, *TP53* [2 variants], and *CHEK2*) (Table S2). In total, we observed ClinVar variants in 18 genes in our cases. However, for 7 of those genes we also observed ClinVar variants in ACpop (*PRF1*, *MYO7A*, *RAD51C*, *BARD1*, *COL7A1*, *SDHA*, and *RECQL4*). Four variants (*PRF1*, *RAD51C*, *COL7A1*, and *SDHA*) were observed in both glioma cases and the healthy reference set ACpop (Table S3).

### Validation of P/LP Variants in Uppsala GBM Individuals

An additional cohort of 105 GBM cases from Uppsala, Sweden (enrolled as part of the U-CAN project site in Uppsala, Sweden) with available WGS variant data was used for validation. We found that 13 cases (12.4%) harbored variants previously described as P/LP in ClinVar, including variants in *SDHA*, *RECQL4*, *FANCA*, *TP53*, *ERCC2*, *CHEK2*, and *MPL*. Interestingly, the same pathogenic variants in *FANCA* and *MPL* were both observed in the Northern Sweden cohort (Table S2). We additionally identified 3 rare coding variants in *ALK* and 1 variant each in *DNMT3A* and *CREBBP*.

### Gene Variant Burden

We performed gene-based burden tests only including rare variants as described in the “Methods” section. In a first analysis, we compared the occurrences of rare coding variants (Figure 2) in 113 sporadic gliomas with the allele frequencies in ACpop. In the burden analysis for all coding variants, 6 genes of 651 showed nominal enrichment (unadjusted *P* < .05) for rare coding variants in our cases, none of which persisted after multiple testing correction: *TP53*, *ALK*, *CREBBP*, *GAS7*, *KMT2A*, and *POU5F1*. *TP53* and *ALK* each harbored 4 variants in the glioma cohort, but none in ACpop. We found 3 variants in *TP53*, of which 1 occurred in 2 individuals. In *ALK*, we found 4 variants, all seen in different individuals (Table S5).

Applying the same analyses to the set of “severe coding variants” (Figure 2), we observed associations for *SMAD4* (*P* = .02), and 5 additional genes with *P* < .1: *PREX2*, *CACNA1D*, *DNMT3A*, *PRSS1*, and *TP53*. Similarly, applying the burden test to the set of “Regulatory variants,” we observed 3 significant genes: *AKAP6*, *CCND3*, and *SSBP2* (all *P* < .05).

To assess the robustness of our analyses using control data from publicly available allele frequencies (ACpop), we generated quantile–quantile (QQ) plots for the gene-based variant burden tests under each filtering criterion (Figure S1). We observed no *P*-value inflation for any of the filtering criteria, but rather a deflation (λ = 0.346-0.547) indicating a potential risk of false negative findings.

### Structural Variants

No observed SV was predicted to have a deleterious effect in any of the 651 genes in the 113 cases, suggesting a limited contribution of SVs to glioma risk in this population.

### Validation in TCGA

We selected genes that either harbored a ClinVar rare variant or had a *P*-value <.1 in our burden tests. Based on these criteria, a total of 49 genes were put forward for validation in TCGA glioma cases. We performed gene-based burden tests using gnomAD allele frequencies as control data. We observed 5 genes (*TP53*, *CREBBP*, *GAS7*, *NBN*, and *STRN*) that showed a higher burden of rare coding variants in the TCGA cases compared to gnomAD (unadjusted *P*-value < .05). Limiting the analyses to severe coding variants identified 2 genes *DNMT3A* and *TP53*, enriched with variants among the TCGA cases (Table S6).

### UKB-Matched Case–Control Study

In UKB, we assessed 49 genes in 833 glioma cases and 8315 matched controls. *TP53* showed a significantly higher (*P* = .005) burden of pLoF and missense variants among glioma cases compared with controls. Thus, *TP53* was the only gene consistently enriched in glioma patients across all 3 datasets (Northern Sweden, TCGA, and UKB). *DNMT3A* showed a borderline significant (*P* = .07) excess burden of pLoF variants among glioma cases and *SMAD4* (*P* = .06) burden of missense + pLoF variants (Table S7).

### Distribution of Rare Variants in TCGA and UKB for CREBBP and DNMT3A

Because our burden analyses indicated enrichment of rare coding variants in *CREBBP* and *DNMT3A*, we next examined whether these variants cluster within specific domains or exons of each gene. We analyzed the distribution of rare coding variants for genes *CREBBP* and *DNMT3A* across glioma cases from TCGA and UKB, comparing them to the control populations in gnomAD and UKB-matched controls. Given the lack of consistent validation in UKB, the following domain-level observations should be seen as exploratory.

For *DNMT3A*, we saw a concentration of potentially protein-disruptive variants, including 2 stop-gained and 1 splice-donor variant in the SAM-dependent methyltransferase (I654-V912) domain, a functional region of the protein (annotated in UniProt). In contrast, such variants were rare in the UKB control group. In TCGA gliomas, we saw no aggregation of pLoF variants in any specific domain but instead observed 3 missense variants in the last exon where missense variants are less frequent in the gnomAD control data (Figure S2a).

For *CREBBP*, we saw no aggregation of rare coding variants in any annotated protein domain. In both TCGA and UKB glioma cases, there was an aggregation of variants in the N- and C-terminal regions. In TCGA, we notably observed a cluster of missense variants in the second exon, where these variants are more uncommon in the gnomAD controls. In UKB, we see the chr16:3850637: G > A missense variant occurring twice in the second exon and 4 missense variants in the last exon of *CREBBP* (Figure S2b).

### Recurrent CREBBP Variant Identified in Multiple Independent Cohorts

We identified a recurrent rare missense variant in *CREBBP* (chr16:3850637: G > A, p. Pro153Leu) in all 4 glioma cohorts. We found 2 individuals in our Swedish samples (1 Northern Sweden and 1 Uppsala patient) corresponding to an allele frequency of 4.6 × 10^−^³, compared to once in the Swedish population dataset SweGen (AF = 5 × 10^−4^). In the TCGA glioma cohort, it was observed 4 times (AF = 2.4 × 10^−^³) compared with an allele frequency of 4.2 × 10^−4^ in gnomAD v2.1.1. Finally, it was observed twice among our UKB glioma cases (AF = 1.2 × 10^−^³) compared to 8 times in the UKB controls (AF = 4.8 × 10^−4^) (Table S8).

### Somatic Mutations in 73 Individuals

For 73 individuals with tumor WGS data, variant calling was performed for SNVs and indels, SVs, and copy number ­alterations. The most common mutated genes were *TP53* (36%), *PTEN* (37%), *TTN* (21%), *NF1* (17%), and *EGFR* (16%). Common SVs identified were *EGFR* amplification (33%), *EGFR*vIII (11%), 1p/19q co-deletion (7%), and *CDKN2B*/*CDKN2A* deletion (29%) (Figure S3).

Out of the 73 individuals with tumor WGS data, 10 carried a P/LP germline variant reported in ClinVar. There was no association between ClinVar P/LP variants and TMB (*P* = .98, Wilcoxon rank test) (Figure S4).

For the 10 individuals where we observed P/LP germline variants, we did not identify any additional somatic mutations. Second hit somatic mutations were found in genes *TERT*, *RHBDF2*, *SOS1*, *SUFU*, and *RB1* (Table S9). These were cases where individuals carried a rare coding germline variant and also had a somatic mutation in the same gene.

## Discussion

In the cohort from Northern Sweden, we found that 17.6% of the individuals harbored a known P/LP variant within 1 of 651 pre-selected genes as compared with 7% of the individuals in the ACpop cohort of individuals from Västerbotten County that reached 80 years without cancer. These results suggest a notable contribution of rare germline variants to glioma risk.

We identified multiple germline ClinVar P/LP variants in several DNA repair genes across the 2 Swedish glioma studies, including *TP53*, *CHEK2*, *FANCA*, *BRCA2*, *RAD51C*, *ERCC2*, *BRIP1*, and *RECQL4*. These results indicate that disruptions in the DNA repair mechanism could play a crucial role in the development of glioma. Notably, rare germline variants in *FANCA* and *RECQL4* have been reported to occur in pediatric glioma patients.^46^ Even though our gene set is enriched with known cancer predisposition genes, it is notable that such a large proportion of our patients held variants in such genes, as compared to the healthy reference population.

A limitation of our study is that we did not apply ACMG guidelines for classification of all variants. Instead, we restricted pathogenicity assignments to variants already listed as P/LP in ClinVar. Applying ACMG classification systematically to all rare variants is challenging in this setting, as it requires extensive manual curation, access to detailed functional and segregation data, and specialized clinical genetics expertise, which were beyond the scope of the present study. This may lead to an underestimation of clinically relevant variants in our cohort.

We observed an enrichment of rare coding variants in *TP53*, *ALK*, *CREBBP*, *GAS7*, *KMT2A*, and *POU5F1*. *TP53* is a well-established tumor suppressor, and its relationship to glioma predisposition has been shown before.^14^^,^^47^^,^^48^ The fact that it showed one of the highest burdens in our study suggests a robustness to our methodology. Furthermore, TP53 was consistently enriched across all 3 patient sets.

Four coding variants were observed in *ALK* and none in the reference population. Although germline and somatic alterations in *ALK*, a tyrosine kinase receptor, are recognized for pediatric neuroblastoma,^49^ it is not recognized in predisposition to glioma. *ALK* is a known predisposition gene for neuroblastoma, where gain-of-function variants have been found to predispose the disease and to play a part in tumor evolution. *ALK* germline variants have been reported in pediatric gliomas,^50^ and germline variants in *ALK* are shown to have a prognostic value in patients with glioma.^51^

*DNMT3A* had a variant burden test consistently higher in our patient cohort and the 2 validation datasets. *DNMT3A* is coding for DNA methyltransferase 3 alpha involved in *de novo* methylation.^52^ Although *DNMT3A* is frequently mutated in acute myeloid leukemia,^53^ its role in gliomagenesis has not been extensively studied. It has been shown that reduced expression of *DNMT3A* in glioma stem cells disrupts the normal *de novo* methylation and is suggested to affect the genomic instability early in glioma ­progression.^54^ Somatic mutations combined in both *DNMT3A* with *IDH1* in astrocytoma grade 4 have further been observed^55^ and have been described as a prognostic marker of LGG.^56^ It is worth noting that clonal hematopoiesis (CHIP) has been shown to result in age-associated *DNMT3A* variants that could be mistaken for germline variants.^57^ Since the ACpop control population all consists of elderly individuals having reached an age of 80,^24^ it is unlikely that the enrichment of *DNMT3A* variants in the Northern Sweden cohort is due to CHIP. Although we think it is unlikely, we cannot rule out that it is due to CHIP.

*CREBBP* showed a high variant burden in cases from northern Sweden and TCGA, but this was not replicated in UKB or in the glioma cases from Uppsala, Sweden. Analysis of variant locations within *CREBBP* showed clustering of coding variants near the first and last exons, a pattern also observed in UKB. One variant, 16:3850637: G > A, is found in higher frequencies in individuals with glioma across all cohorts. *CREBBP*, coding for the acetyltransferase CREB-binding protein, is involved in chromatin remodeling, which plays a considerable role in neurodevelopment.^58^ Somatic mutations in *CREBBP* have been reported in medulloblastoma,^59^ but are uncommon in glioma. Deletion of *CREBBP* together with overexpression of *MYCN* have been shown to induce the formation of brain tumors in mice, suggesting a potential role in gliomagenesis.^60^ Moreover *CREBBP* has been suggested to be involved in driving H2B acetylation in GBM.^61^ Given the CHIP susceptibility of DNMT3A and the absence of consistent replication, we avoid nominating *DNMT3A* and *CREBBP* as predisposition genes, but believe they are plausible genes for further follow-up studies.

The results from this study showed little overlap with results from similar studies; no single rare deleterious variants nor higher gene variant burden compared to the reference population were found in previously implicated genes such as *HERC2*, *MYO7A*, *IP6K1*, *ADAMTS8*, and *LRRK2.*^22^ Although previous studies of familial gliomas have pointed to co-segregation of rare deleterious variants in *POT1*^20^ and *POLE*,^21^ no deleterious variants in these genes were found in this study. However, we observed a splice acceptor variant in 1 individual in *POLD1* (Table S4). A possible explanation is that previous studies were performed on familial gliomas, whereas our focus is on sporadic cases without a known family history. We observed no deleterious variants in mismatch repair genes *MLH1*, *MSH2*, *MSH6*, and *PMS2* in our Northern Swedish cohort, but 2 deleterious variants in *MSH2* and *MSH6* were observed among our cases enrolled in Uppsala, in agreement with previous studies.^12^^,^^13^^,^^62^^,^^63^

Interestingly, several of the genes highlighted in our analyses, including *CREBBP*, *DNMT3A*, and *KMT2A*, are not recognized as cancer predisposition genes but are instead associated with autosomal dominant developmental ­syndromes. Pathogenic variants in *CREBBP* cause Rubinstein–Taybi syndrome (RTS), a condition characterized by intellectual disability and congenital anomalies, and RTS has been linked to increased tumor risk, including brain tumors.^64^ Similarly, *DNMT3A* is the causal gene for Tatton-Brown–Rahman syndrome (TBRS), an overgrowth disorder. This phenotype has been associated with an elevated risk for hematopoietic malignancies.^65^ In this study, we did not have access to detailed clinical information on additional diagnoses such as RTS or TBRS syndrome in our cohort and therefore cannot determine whether any of the carriers of these variants exhibited these phenotypes. Nevertheless, these observations highlight that some rare germline variants identified in glioma patients may reflect broader developmental or genetic syndromes, not all of which are necessarily causal for glioma itself. Future studies integrating genetic and clinical phenotype data will be important to disentangle whether variants in such genes contribute directly to glioma predisposition or represent coincidental findings related to underlying Mendelian conditions.

We did not find an association between germline rare P/LP variants and somatic mutation patterns in tumor or in the TMB of these individuals. This may stem from our limited sample size of sequenced tumors and subsequently small number who carried P/LP germline variants. It is possible that germline variants predisposing to glioma does so by influencing early tumor initiation, such as DNA repair, and have no further subsequent effect on the development of the disease. Future studies in larger cohorts that integrate germline and tumor data are needed to clarify any relationships between germline and somatic alterations.

One limitation of this study is the relatively small sample size of our initial discovery cohort (*n* = 113). In addition, different sequencing chemistries and different bioinformatic workflows were used across the analyzed datasets. This could introduce biases in the coverage and quality of variant calling between the datasets, limiting our ability to validate our findings. It has been shown that leveraging public datasets as controls can inflate the false-positive discovery.^66^ Ideally, future studies would use fully harmonized sequencing and analytic workflows for all datasets. To overcome these limitations, we have leveraged publicly available allele frequencies from different resources and further validated our findings within a more robust epidemiological context using large-scale UKB data. As such, we have reported the unadjusted *P*-values in the results, although no result would reach a significant threshold of *P* < .05 after accounting for multiple testing. As we evaluated 651 genes in our Northern Sweden discovery cohort, the use of an unadjusted *P*-value threshold of .10 as a discovery filter implies that approximately 65 genes would be expected to meet this cutoff by chance. When filtering for all coding variants, the burden tests yielded 33 genes with a *P*-value < .1; as such, we get fewer hits than expected, which is also implicated by the QQ-plots. The *P* < .1 threshold should therefore be seen as hypothesis-generating, rather than evidence of association.

Despite the limited sample size, our study shows that 20 of 113, that is, every sixth glioma patient (17.6%), harbor a P/LP germline variant within the selected genes. This may be important information in the selection of therapeutic targets for glioma therapy and also displays the complexity and low frequency of the variants per gene. This calls for an approach of very specified selection of small glioma subsets into clinical trials when evaluating response to therapy. Validation in an independent cohort from Uppsala Sweden identified additional pathogenic variants in 6 of these genes. These results alone suggest a significant contribution of rare germline variants to glioma risk.

## Supplementary Material

vdag038_Supplementary_Data

## Data Availability

Aggregated variant data can be made available upon reasonable request and appropriate approvals are in place. Variant data will only be shared such that individual-level information can not be inferred. Due to privacy regulations, raw sequencing data will not be made publicly available.
