# Supplementary material for: Rare germline variants contribute to glioma predisposition: Whole-genome analysis of a regional cohort of glioma patients
Source: Neurooncol Adv. 2026 Feb 12;8(1):vdag038. doi: 10.1093/noajnl/vdag038 (PMC13007284; doi:10.1093/noajnl/vdag038)
Supplement: vdag038_Supplementary_Data [file vdag038_supplementary_data.zip › Supplementary_Figures.docx]

## Figure S1: Figure S1. Quantile-Quantile plots p-values from gene-wise variant burden tests

QQ-plots showing the distribution of p-values from gene-wise burden tests using different filtering criteria: coding variants (top), high-impact variants (middle) and regulatory variants (bottom). Observed p-values for each gene on the y-axis, with expected p-values on x-axis. The inflation factor (lambda) is displayed in red for each filtering strategy.


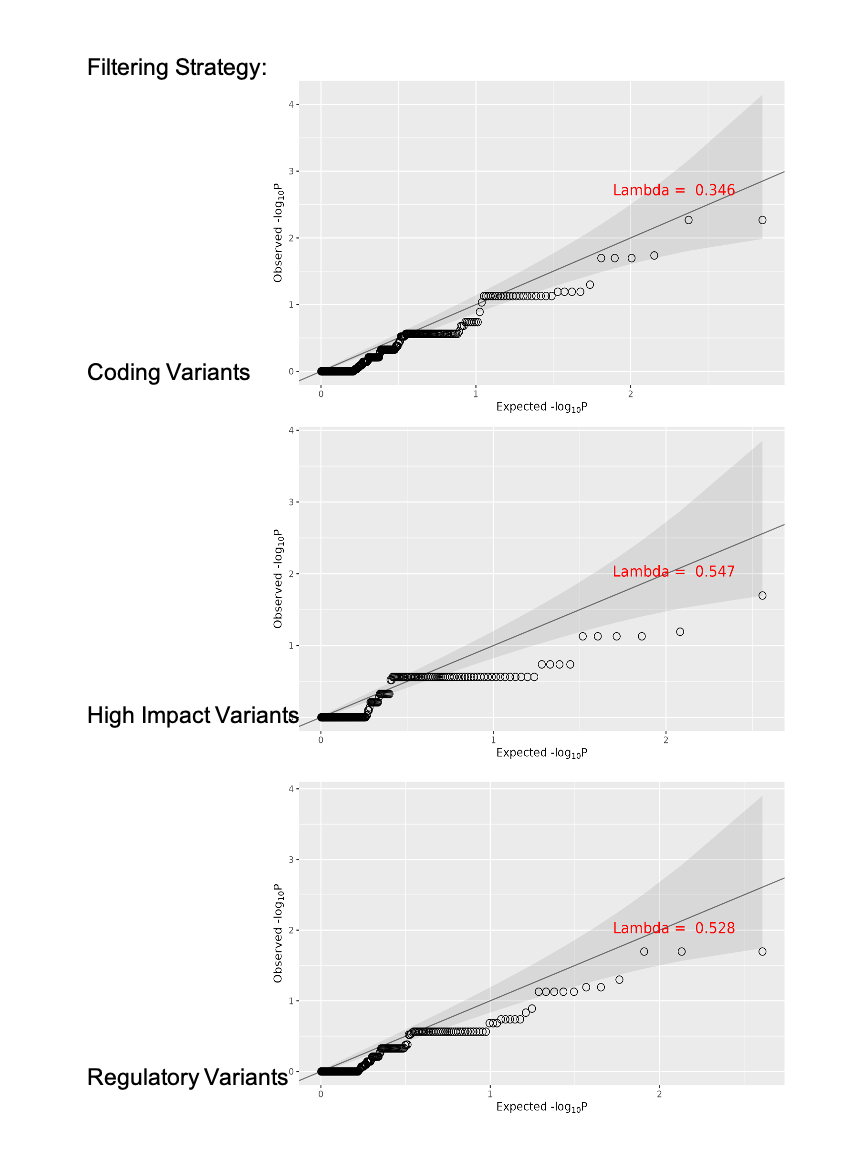


## Figure S2: Distribution of Rare Coding Variants in *DNMT3A* and *CREBBP* across TCGA and UKBB glioma cases.

Rare coding variant allele frequencies observed in TCGA and UKBB glioma case sets (top panel of diagram) compared to respective control frequencies in gnomAD, and UKBB matched controls (lower panel). UniProt domains overlap gene exons for both *DNMT3A* (a) and *CREBBP* (b).

##
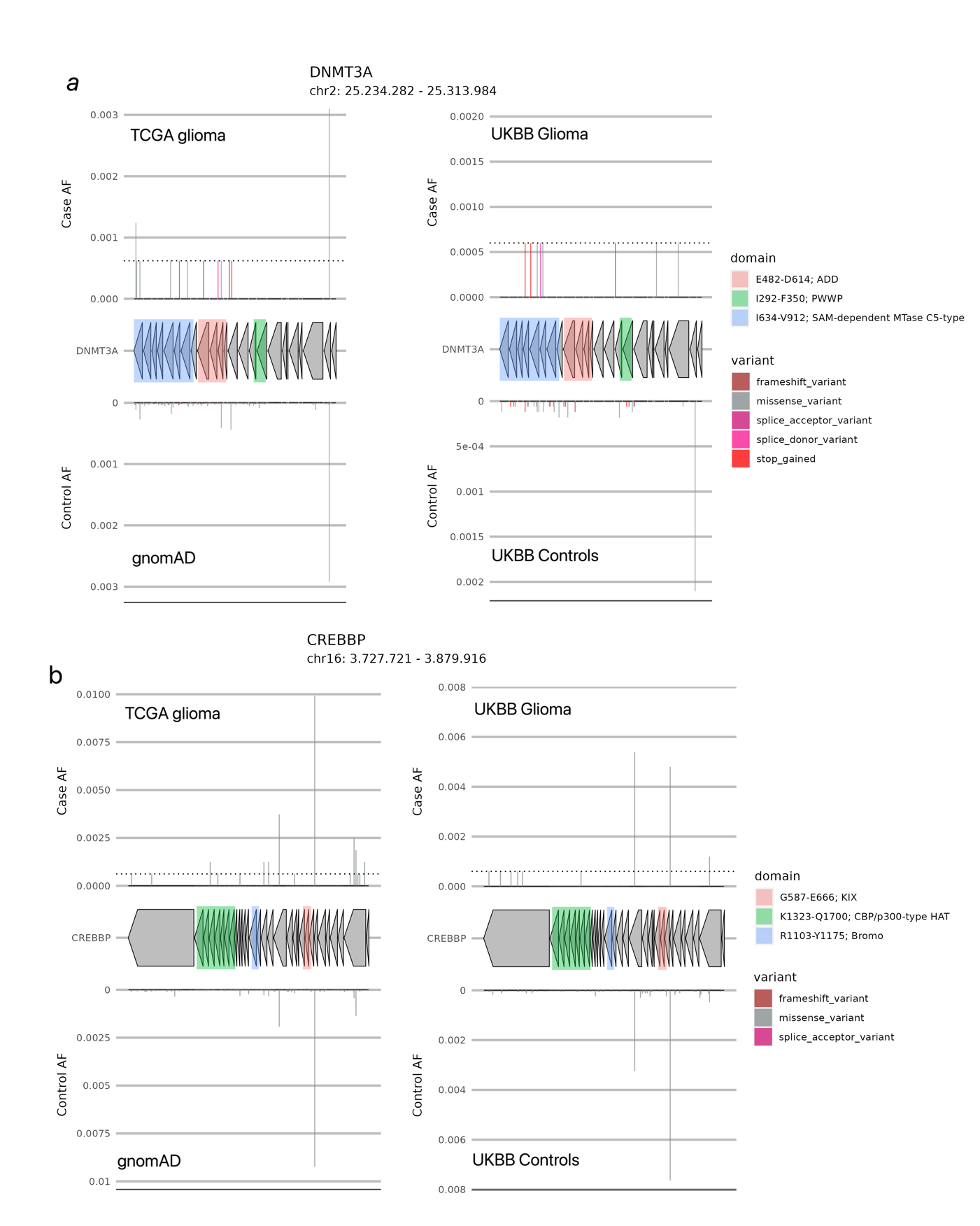


## Figure S3: Somatic Alterations in 73 Glioma Cases.

Oncoprint with occurrences of somatic alteration (rows) for 73 glioma cases (columns), highlighting differences between patients with (black bars) and without pathogenic ClinVar variants.

##
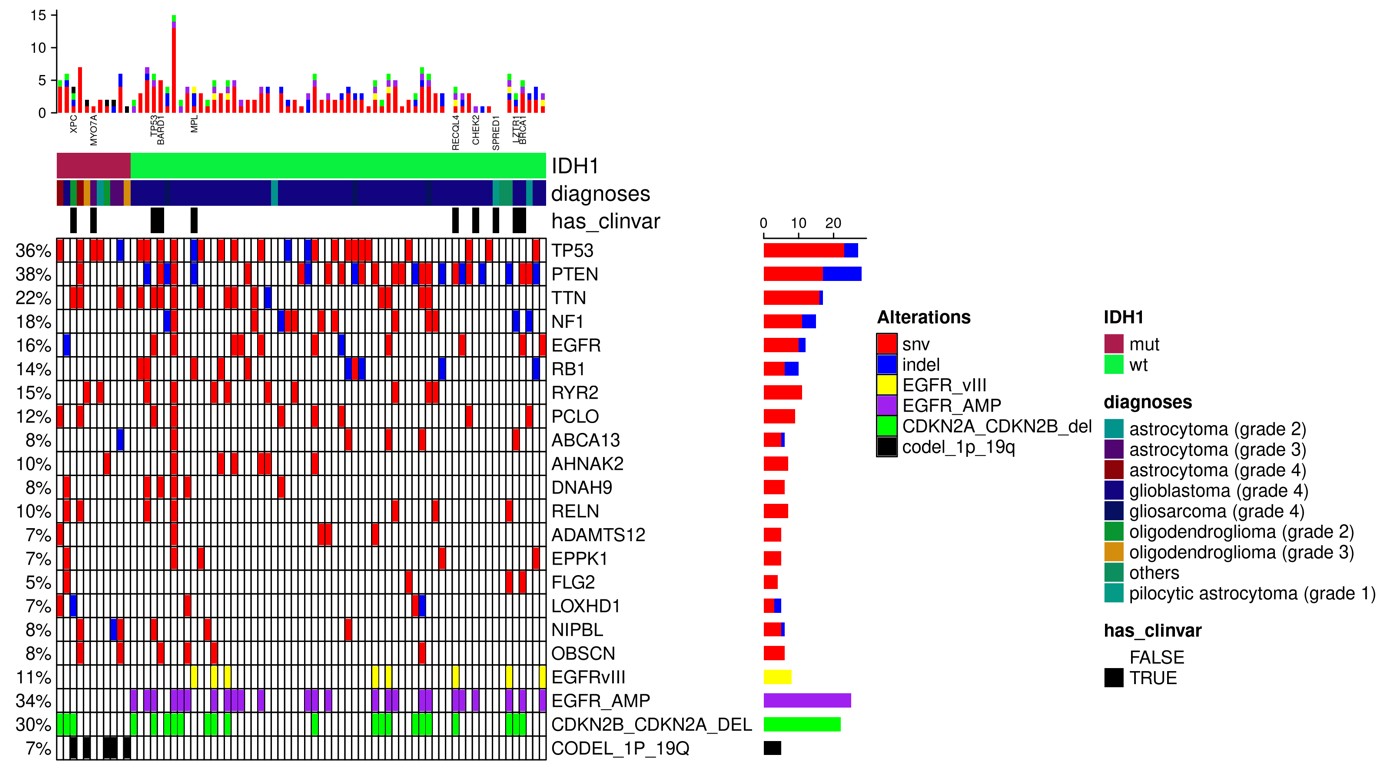


## Figure S4: Tumor Mutational Burden (TMB) in 73 glioma Cases with and without Pathogenic Germline Variants


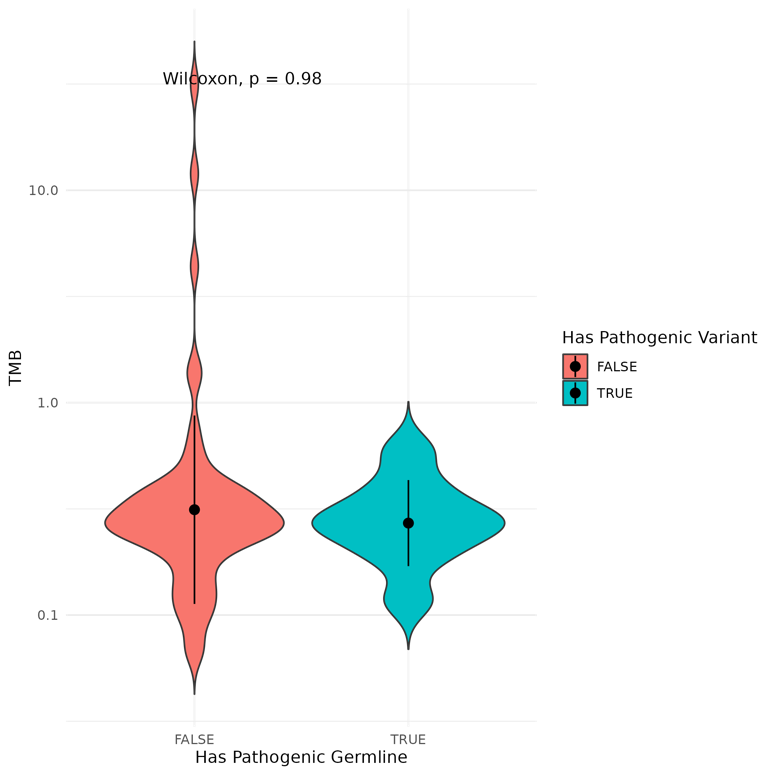
Violin plots comparing the tumor mutational burden (TMB) in glioma cases based on the presence (blue) and absence (red) of pathogenic or likely pathogenic ClinVar germline variants. The median and interquartile range for each group is indicated by black dots and lines.
