# Supplementary material for: Rare germline variants contribute to glioma predisposition: Whole-genome analysis of a regional cohort of glioma patients
Source: Neurooncol Adv. 2026 Feb 12;8(1):vdag038. doi: 10.1093/noajnl/vdag038 (PMC13007284; doi:10.1093/noajnl/vdag038)
Supplement: vdag038_Supplementary_Data [file vdag038_supplementary_data.zip › Table_S2-S9.docx]

## Table S2: Overview of patient sets used in study

| Cohort / Dataset | Cases (n) | Controls (n) | Sequencing / Source | Sample type(s) | Key inclusion/notes | Role in study |
| --- | --- | --- | --- | --- | --- | --- |
| Northern Sweden glioma cohort | 113 | – | WGS (blood for all; tumor for subset) | Germline blood (113); Tumor tissue (73) | Two partially overlapping groups: Early-onset 18–40 y (n=46) and Tumor-Germline (n=73; 6 overlap). Sequenced 30× germline; 90× tumor (NovaSeq for TG; HiSeqX for EO). | Discovery cohort. |
| Uppsala glioblastoma cohort | 105 | – | WGS germline calls | Germline blood | GBM only; processed with nf-core/sarek; HiSeqX, TruSeq PCR-free, ≥30×. | Validation of P/LP |
| TCGA glioma (European ancestry subset) | 806 | – | Germline calls from (WES); harmonized | Germline (normal) | Low- and high-grade glioma; European subset from 10,389 TCGA germlines described by Huang et al. Here 335 GBM and 471 LGG were used. | Validation (burden vs gnomAD). |
| UK Biobank glioma case–control | 833 | 8,315 | WES | Germline | European ancestry; ICD-coded glioma; ~1:10 age-matched controls; REGENIE burden on 49 genes. | Validation (case–control burden). |

## Table S3: P/LP variants in Northern Sweden, Uppsala, and ACpop

| P/LP Clinvar Variants in Northern Sweden | | | | | | |  | |  |  |  |  |  |  |  |  |  |
| --- | --- | --- | --- | --- | --- | --- | --- | --- | --- | --- | --- | --- | --- | --- | --- | --- | --- |
| **Position** | **HGNCp** | **Clinvar Entry** | **Swegen freq** | **Gene** | **CADD score** | **AlphaMissense** | | **Consequence (VEP)** | **cosmic_entries** | **Alleles** | **Diagnosis** | **IDH-status** | **cosmic_entries** | **Variant in Acpop** | **Gene has variant in Acpop** | **Variant in Uppsala Cohort** | **Gene in Uppsala Cohort** |
| **chr1:43338634:G:C** | p.Arg102Pro | Pathogenic | 0 | MPL | 26.3 | likely_pathogenic | | missense_variant | 1 | 1 | glioblastoma (grade 4) | wt | 1 | false | false | true | true |
| **chr2:214781360:C:CT** | p.Asp172ArgfsTer10 | Pathogenic | 0 | BARD1 | 19.22 | . | | frameshift_variant | 0 | 1 | glioblastoma (grade 4) | wt | 0 | false | true | false | false |
| **chr3:14148560:A:G** | . | Likely_pathogenic | 0 | XPC | 34 | . | | splice_donor_variant | 0 | 1 | oligodendroglioma (grade 2) | mut | 0 | false | false | false | false |
| **chr3:48572138:G:A** | p.Arg2338Ter | Pathogenic | 0.0005 | COL7A1 | 35 | . | | stop_gained | 0 | 1 | astrocytoma (grade 4) | mut | 0 | true | true | false | false |
| **chr5:224432:C:T** | p.Arg75Ter | Pathogenic/Likely_pathogenic | 0.0005 | SDHA | 37 | . | | stop_gained | 1 | 1 | astrocytoma (grade 2) | mut | 1 | true | true | false | true |
| **chr7:142751919:C:T** | p.Arg116Cys | Pathogenic/Likely_pathogenic | 0 | PRSS1 | 19.02 | likely_pathogenic | | missense_variant | 1 | 1 | astrocytoma (grade 2) | wt | 1 | false | false | false | false |
| **chr8:144513412:G:A** | p.Gln757Ter | Pathogenic | 0 | RECQL4 | 39 | . | | stop_gained | 0 | 1 | glioblastoma (grade 4) | wt | 0 | false | true | false | true |
| **chr8:144515891:C:T** | . | Likely_pathogenic | 0 | RECQL4 | 23.3 | . | | splice_acceptor_variant | 0 | 1 | astrocytoma (grade 2) | mut | 0 | false | true | false | true |
| **chr9:97675579:G:A** | p.Arg228Ter | Pathogenic | 0.0005 | XPA | 41 | . | | stop_gained | 0 | 1 | oligodendroglioma (grade 2) | mut | 0 | false | false | false | false |
| **chr10:70598772:C:T** | p.Gly317Arg | Pathogenic | 0.0005 | PRF1 | 22.4 | ambiguous | | missense_variant | 0 | 1 | glioblastoma (grade 4) | wt | 0 | true | true | false | false |
| **chr11:77156022:T:A** | p.Ile134Asn | Likely_pathogenic | 0 | MYO7A | 28.4 | likely_pathogenic | | missense_variant | 0 | 1 | astrocytoma (grade 3) | mut | 0 | false | true | false | false |
| **chr15:38351477:CAGAG:C** | p.Gly385IlefsTer20 | Pathogenic/Likely_pathogenic | 0 | SPRED1 | . | . | | frameshift_variant | 0 | 1 | pilocytic astrocytoma (grade 1) | wt | 0 | false | false | false | false |
| **chr16:89815966:T:A** | p.Lys34Ter | Pathogenic | 0.0005 | FANCA | 35 | . | | stop_gained | 0 | 1 | astrocytoma (grade 2) | mut | 0 | false | false | true | true |
| **chr17:7674972:C:T** | . | Pathogenic | 0 | TP53 | 33 | . | | splice_acceptor_variant | 4 | 1 | glioblastoma (grade 4) | wt | 4 | false | false | false | true |
| **chr17:7675139:C:T** | p.Arg158His | Pathogenic/Likely_pathogenic | 0 | TP53 | 23.3 | likely_pathogenic | | missense_variant | 4 | 1 | glioblastoma (grade 4) | wt | 4 | false | false | false | true |
| **chr17:43093514:C:A** | p.Glu673Ter | Pathogenic | 0 | BRCA1 | 35 | . | | stop_gained | 2 | 1 | glioblastoma (grade 4) | wt | 2 | false | false | false | false |
| **chr17:58709926:GT:G** | p.Thr259LeufsTer4 | Pathogenic/Likely_pathogenic | 0.0005 | RAD51C | . | . | | frameshift_variant | 0 | 1 | glioblastoma (grade 4) | wt | 0 | true | true | false | false |
| **chr19:45352801:C:G** | p.Arg616Pro | Pathogenic | 0 | ERCC2 | 28.7 | likely_pathogenic | | missense_variant | 1 | 1 | astrocytoma (grade 2) | mut | 1 | false | false | false | true |
| **chr22:20993977:G:A** | p.Trp469Ter | Pathogenic | 0 | LZTR1 | 44 | . | | stop_gained | 0 | 1 | glioblastoma (grade 4) | wt | 0 | false | false | false | false |
| **chr22:28725338:T:C** | p.Arg117Gly | Pathogenic/Likely_pathogenic | 0.0005 | CHEK2 | 26.1 | likely_pathogenic | | missense_variant | 1 | 1 | glioblastoma (grade 4) | wt | 1 | false | false | false | true |

P/LP Clinvar Variants in ACpop

| **Postition** | **HGNCp** | **Clinvar Entry** | **Swegen freq** | **Gene** | **CADD score** | **AlphaMissense** | **Consequence** | **Alleles** |
| --- | --- | --- | --- | --- | --- | --- | --- | --- |
| chr2:47799329:T:C | p.Leu449Pro | Pathogenic | 0 | MSH6 | 28.3 | likely_pathogenic | missense_variant | 1 |
| chr2:127272934:CT:C | p.Gln586ArgfsTer25 | Pathogenic/Likely_pathogenic | 0.0005 | ERCC3 | 33 | . | frameshift_variant | 1 |
| chr2:214728708:TCA:T | p.Val767AspfsTer4 | Pathogenic/Likely_pathogenic | 0 | BARD1 | 32 | . | frameshift_variant | 1 |
| chr3:48572138:G:A | p.Arg2338Ter | Pathogenic | 0.0005 | COL7A1 | 35 | . | stop_gained | 1 |
| chr5:224432:C:T | p.Arg75Ter | Pathogenic/Likely_pathogenic | 0.0005 | SDHA | 37 | . | stop_gained | 4 |
| chr8:144516069:CCT:C | p.Arg350GlyfsTer21 | Pathogenic/Likely_pathogenic | 0 | RECQL4 | 17.03 | . | frameshift_variant | 2 |
| chr10:70598772:C:T | p.Gly317Arg | Pathogenic | 0.0005 | PRF1 | 22.4 | ambiguous | missense_variant | 2 |
| chr11:77174825:C:T | p.Arg669Ter | Pathogenic/Likely_pathogenic | 0.0005 | MYO7A | 37 | . | stop_gained | 2 |
| chr12:27647774:C:T | p.Arg135Ter | Pathogenic | 0.0005 | PPFIBP1 | 43 | . | stop_gained | 1 |
| chr14:92004014:A:T | p.Leu1321Ter | Likely_pathogenic | 0 | TRIP11 | 33 | . | stop_gained | 3 |
| chr15:80168263:G:T | . | Pathogenic | 0 | FAH | 33 | . | splice_acceptor_variant | 1 |
| chr16:23626291:C:T | p.Trp898Ter | Pathogenic | 0 | PALB2 | 40 | . | stop_gained | 1 |
| chr17:58709926:GT:G | p.Thr259LeufsTer4 | Pathogenic/Likely_pathogenic | 0.0005 | RAD51C | . | . | frameshift_variant | 2 |

|  | |  | | P/LP Clinvar variants in Uppsala | | | | | | | | |
| --- | --- | --- | --- | --- | --- | --- | --- | --- | --- | --- | --- | --- |
| **Position** | **HGNCp** | | **Clinvar Entry** | | **Swegen freq** | **Gene** | **CADD score** | **Cosmic entries** | **AlphaMissense** | **Consequence** | **Alleles** | **Diagnosis** |
| chr1:43338634:G:C | p.Arg102Pro | | Pathogenic | | 0 | MPL | 26,3 | 1 | likely_pathogenic | missense_variant | 1 | astrocytoma (who grade 4) |
| chr1:149002952:C:T | p.Arg1292Cys | | Likely_pathogenic | | 0 | PDE4DIP | 23,8 | 1 | likely_benign | missense_variant | 1 | glioblastoma (who grade 4) |
| chr2:47475050:CAAT:C | p.Asn596del | | Pathogenic | | 0 | MSH2 | . | 0 | . | inframe_deletion | 1 | oligodendroglioma (who grade 3) |
| chr2:47803500:A:AC | p.Phe1088LeufsTer5 | | Pathogenic | | 0 | MSH6 | 24,9 | 0 | . | frameshift_variant | 1 | astrocytoma (who grade 4) |
| chr2:168924745:C:A | p.Arg1226Leu | | Likely_pathogenic | | 0,0005 | ABCB11 | 29,1 | 0 | likely_pathogenic | missense_variant | 1 | glioblastoma (who grade 4) |
| chr5:223509:C:T | p.Arg31Ter | | Pathogenic/Likely_pathogenic | | 0,0005 | SDHA | 34 | 1 | . | stop_gained | 1 | glioblastoma (who grade 4) |
| chr8:144512325:C:T | . | | . | | 0 | RECQL4 | 22,9 | 0 | . | splice_acceptor_variant | 1 | glioblastoma (who grade 4) |
| chr16:89815966:T:A | p.Lys34Ter | | Pathogenic | | 0,0005 | FANCA | 35 | 0 | . | stop_gained | 1 | glioblastoma (who grade 4) |
| chr17:7675185:C:T | p.Val143Met | | Conflicting_interpretations_of_pathogenicity | | 0 | TP53 | 24,1 | 4 | likely_pathogenic | missense_variant | 1 | glioblastoma (who grade 4) |
| chr17:61686008:C:CA | p.Thr912AspfsTer27 | | Pathogenic | | 0 | BRIP1 | 17,47 | 0 | . | frameshift_variant | 1 | glioblastoma (who grade 4) |
| chr19:45365053:G:A | p.Arg156Ter | | Pathogenic | | 0 | ERCC2 | 40 | 1 | . | stop_gained | 1 | glioblastoma (who grade 4) |
| chr22:19234688:C:T | p.Glu330Lys | | Likely_pathogenic | | 0,0005 | CLTCL1 | 23,7 | 0 | likely_benign | missense_variant | 1 | glioblastoma (who grade 4) |
| chr22:28734401:A:T | . | | Pathogenic/Likely_pathogenic | | 0 | CHEK2 | 34 | 0 | . | splice_donor_variant | 1 | glioblastoma (who grade 4) |

## Table S4: LoF variants in Northern Sweden

| position | HGNCp | swegen_freq | gene | CADD score | cosmic_entries | consequence | cosmic_entries.1 | num_alleles | Diagnosis | idh_status | age_below_40 | gene pli > 0.9 |
| --- | --- | --- | --- | --- | --- | --- | --- | --- | --- | --- | --- | --- |
| chr1:148978372:TA:T | p.Glu911AsnfsTer17 | . | PDE4DIP | 23.1 | 0 | frameshift_variant | 0 | 1 | astrocytoma (grade 2) | mut | TRUE | FALSE |
| chr1:149005330:G:GGACCT | p.Glu1505AspfsTer21 | 0.0005 | PDE4DIP | 32 | 0 | frameshift_variant | 0 | 1 | glioblastoma (grade 4) | wt | FALSE | FALSE |
| chr2:25234311:CG:C | p.Phe902LeufsTer4 | . | DNMT3A | 28.9 | 1 | frameshift_variant | 1 | 1 | astrocytoma (grade 2) | mut | FALSE | FALSE |
| chr2:29223524:G:C | p.Tyr1059Ter | . | ALK | 46 | 0 | stop_gained | 0 | 1 | glioblastoma (grade 4) | wt | FALSE | FALSE |
| chr2:214781360:C:CT | p.Asp172ArgfsTer10 | . | BARD1 | 19.22 | 0 | frameshift_variant | 0 | 1 | glioblastoma (grade 4) | wt | FALSE | FALSE |
| chr2:215319684:TC:T | p.Pro82GlnfsTer15 | . | ATIC | 27.8 | 1 | frameshift_variant | 1 | 1 | glioblastoma (grade 4) | wt | FALSE | FALSE |
| chr3:14148560:A:G | . | . | XPC | 34 | 0 | splice_donor_variant | 0 | 1 | oligodendroglioma (grade 2) | mut | FALSE | FALSE |
| chr3:48572138:G:A | p.Arg2338Ter | 0.0005 | COL7A1 | 35 | 0 | stop_gained | 0 | 1 | astrocytoma (grade 4) | mut | TRUE | FALSE |
| chr4:105275131:C:T | p.Gln1541Ter | . | TET2 | 35 | 1 | stop_gained | 1 | 1 | glioblastoma (grade 4) | wt | FALSE | FALSE |
| chr5:224432:C:T | p.Arg75Ter | 0.0005 | SDHA | 37 | 1 | stop_gained | 1 | 1 | astrocytoma (grade 2) | mut | TRUE | FALSE |
| chr7:75559730:A:C | . | . | HIP1 | 33 | 0 | splice_donor_variant | 0 | 1 | astrocytoma (grade 2) | unknown | TRUE | FALSE |
| chr7:152163399:TCACTA:T | p.Phe3391LeufsTer9 | . | KMT2C | . | 0 | frameshift_variant | 0 | 1 | astrocytoma (grade 2) | mut | FALSE | TRUE |
| chr8:68108332:G:T | . | . | PREX2 | 34 | 0 | splice_donor_variant | 0 | 1 | glioblastoma (grade 4) | wt | FALSE | FALSE |
| chr8:129399686:T:G | . | . | CCDC26 | 10.98 | 0 | splice_acceptor_variant&non_coding_transcript_variant | 0 | 1 | gliosarcoma (grade 4) | wt | FALSE | FALSE |
| chr8:133239012:G:A | p.Arg351Ter | . | NDRG1 | 44 | 0 | stop_gained | 0 | 1 | oligodendroglioma (grade 2) | mut | TRUE | FALSE |
| chr8:144513412:G:A | p.Gln757Ter | . | RECQL4 | 39 | 0 | stop_gained | 0 | 1 | glioblastoma (grade 4) | wt | FALSE | FALSE |
| chr8:144515891:C:T | . | . | RECQL4 | 23.3 | 0 | splice_acceptor_variant | 0 | 1 | astrocytoma (grade 2) | mut | TRUE | FALSE |
| chr9:97675579:G:A | p.Arg228Ter | 0.0005 | XPA | 41 | 0 | stop_gained | 0 | 1 | oligodendroglioma (grade 2) | mut | TRUE | FALSE |
| chr11:69643118:AAG:A | p.Ser97ProfsTer15 | . | CCND1 | . | 0 | frameshift_variant | 0 | 1 | glioblastoma (grade 4) | wt | FALSE | FALSE |
| chr11:119274831:G:A | . | . | CBL | 35 | 1 | splice_acceptor_variant | 1 | 1 | glioblastoma (grade 4) | wt | FALSE | FALSE |
| chr12:58890771:G:GT | p.Leu137ThrfsTer3 | . | LRIG3 | . | 0 | frameshift_variant | 0 | 1 | astrocytoma (grade 3) | mut | TRUE | FALSE |
| chr12:122278940:GACCTGAAACACAGTTGTTTAGCTTAGGCTGAGGGTTTGAACGAAAGGAGGCCGCGTGAAAGCTCGTGCACCCTGGGTGGTACTCT:G | . | . | CLIP1 | . | 0 | splice_acceptor_variant&splice_donor_5th_base_variant&coding_sequence_variant&intron_variant | 0 | 1 | oligodendroglioma (grade 2) | mut | TRUE | FALSE |
| chr12:122319348:C:CTTTGGCTTTGTCGGCTTGCTTTCTCAGCTCCTCCAGCTCCTGCAGCAAGCCACTGTTCTCCTCCCGTGCG | p.Ala1084ArgfsTer44 | . | CLIP1 | . | 0 | frameshift_variant&splice_region_variant | 0 | 1 | oligodendroglioma (grade 2) | mut | TRUE | FALSE |
| chr12:122328161:TCCTGCAGAGACCCCGAATGAGGGAATGAGTCATCTGCCCATGCGTGTACTATCCCTTGCCTTCCTTGCCAGCCAACAGGCCCACTGAGTATCTCCTTA:T | . | . | CLIP1 | . | 0 | splice_acceptor_variant&splice_donor_variant&splice_donor_5th_base_variant&coding_sequence_variant&intron_variant | 0 | 1 | oligodendroglioma (grade 2) | mut | TRUE | FALSE |
| chr15:38351477:CAGAG:C | p.Gly385IlefsTer20 | . | SPRED1 | . | 0 | frameshift_variant | 0 | 1 | pilocytic astrocytoma (grade 1) | wt | TRUE | TRUE |
| chr15:50481712:G:T | p.Glu484Ter | . | USP8 | 37 | 0 | stop_gained | 0 | 1 | astrocytoma (grade 4) | mut | FALSE | TRUE |
| chr16:3728478:CG:C | p.Arg2190GlufsTer112 | . | CREBBP | . | 1 | frameshift_variant | 1 | 1 | glioblastoma (grade 4) | wt | FALSE | TRUE |
| chr16:23603548:GT:G | p.Gln1157HisfsTer6 | . | PALB2 | . | 0 | frameshift_variant | 0 | 1 | glioblastoma (grade 4) | wt | FALSE | FALSE |
| chr16:89815966:T:A | p.Lys34Ter | 0.0005 | FANCA | 35 | 0 | stop_gained | 0 | 1 | astrocytoma (grade 2) | mut | TRUE | FALSE |
| chr17:7674972:C:T | . | . | TP53 | 33 | 4 | splice_acceptor_variant | 4 | 1 | glioblastoma (grade 4) | wt | FALSE | FALSE |
| chr17:8143560:ATAGT:A | p.Asn925IlefsTer196 | . | PER1 | 32 | 0 | frameshift_variant | 0 | 1 | astrocytoma (grade 2) | mut | TRUE | FALSE |
| chr17:39470877:A:G | . | . | CDK12 | 24.5 | 0 | splice_acceptor_variant | 0 | 1 | astrocytoma (grade 3) | mut | FALSE | TRUE |
| chr17:43093514:C:A | p.Glu673Ter | . | BRCA1 | 35 | 2 | stop_gained | 2 | 1 | glioblastoma (grade 4) | wt | FALSE | FALSE |
| chr17:58709926:GT:G | p.Thr259LeufsTer4 | 0.0005 | RAD51C | . | 0 | frameshift_variant | 0 | 1 | glioblastoma (grade 4) | wt | FALSE | FALSE |
| chr17:78995191:T:TCCTTCACTGCCAGCCATTCGGCCTTGAAGCCTGGC | p.Asp221GlyfsTer11 | . | CANT1 | . | 0 | stop_gained&frameshift_variant | 0 | 1 | astrocytoma (grade 2) | wt | FALSE | FALSE |
| chr18:51058244:AGTATGTACATACTTTAAAAAATCTTTTAAATAGTTGAGAAAAAAGTAGGCAGCCTTTATAAAAGCAAATTAACCCATGTGGGCCTTAATTTTTAG:A | . | 0.0005 | SMAD4 | . | 0 | splice_acceptor_variant&splice_donor_variant&splice_donor_5th_base_variant&splice_polypyrimidine_tract_variant&intron_variant | 0 | 1 | glioblastoma (grade 4) | wt | FALSE | TRUE |
| chr18:58696364:A:T | . | . | MALT1 | 35 | 1 | splice_acceptor_variant | 1 | 1 | glioblastoma (grade 4) | wt | FALSE | TRUE |
| chr19:50401769:ACCCCACAGGCCCAGCGCAGCCTGTGCCTGGGGGGC:A | . | . | POLD1 | . | 0 | splice_acceptor_variant&coding_sequence_variant&intron_variant | 0 | 1 | others | wt | FALSE | FALSE |
| chr22:20993977:G:A | p.Trp469Ter | . | LZTR1 | 44 | 0 | stop_gained | 0 | 1 | glioblastoma (grade 4) | wt | FALSE | FALSE |

## Table S5: Results from gene-wise variant burden tests for the 3 filtering strategies, coding variants, severe coding variants, and regulatory variants. All genes with a p-value < 0.1 is displayed.

| **coding variants** | | | | | |
| --- | --- | --- | --- | --- | --- |
| **Gene** | **CaseOccurences** | **ControlOccurences** | **CaseTotal** | **ControlTotal** | **p_value** |
| ALK | 4 | 0 | 113 | 300 | 0,0054 |
| TP53 | 4 | 0 | 113 | 300 | 0,0054 |
| CREBBP | 5 | 2 | 113 | 300 | 0,0184 |
| GAS7 | 3 | 0 | 113 | 300 | 0,0201 |
| KMT2A | 3 | 0 | 113 | 300 | 0,0201 |
| POU5F1 | 3 | 0 | 113 | 300 | 0,0201 |
| NUP98 | 4 | 2 | 113 | 300 | 0,0502 |
| FGFR3 | 3 | 1 | 113 | 300 | 0,0642 |
| SMAD4 | 3 | 1 | 113 | 300 | 0,0642 |
| SND1 | 3 | 1 | 113 | 300 | 0,0642 |
| WDR7 | 3 | 1 | 113 | 300 | 0,0642 |
| ABCB11 | 2 | 0 | 113 | 300 | 0,0744 |
| AFF4 | 2 | 0 | 113 | 300 | 0,0744 |
| BRD4 | 2 | 0 | 113 | 300 | 0,0744 |
| CACNA1D | 2 | 0 | 113 | 300 | 0,0744 |
| CBL | 2 | 0 | 113 | 300 | 0,0744 |
| CCDC6 | 2 | 0 | 113 | 300 | 0,0744 |
| CEBPA | 2 | 0 | 113 | 300 | 0,0744 |
| CUX1 | 2 | 0 | 113 | 300 | 0,0744 |
| DICER1 | 2 | 0 | 113 | 300 | 0,0744 |
| ERBB3 | 2 | 0 | 113 | 300 | 0,0744 |
| JAK2 | 2 | 0 | 113 | 300 | 0,0744 |
| MITF | 2 | 0 | 113 | 300 | 0,0744 |
| NBN | 2 | 0 | 113 | 300 | 0,0744 |
| NSD1 | 2 | 0 | 113 | 300 | 0,0744 |
| PDGFRA | 2 | 0 | 113 | 300 | 0,0744 |
| PRSS1 | 2 | 0 | 113 | 300 | 0,0744 |
| RB1 | 2 | 0 | 113 | 300 | 0,0744 |
| RUNX1 | 2 | 0 | 113 | 300 | 0,0744 |
| STRN | 2 | 0 | 113 | 300 | 0,0744 |
| XPA | 2 | 0 | 113 | 300 | 0,0744 |
| ZC3H7B | 2 | 0 | 113 | 300 | 0,0744 |
| COL7A1 | 4 | 3 | 113 | 300 | 0,0926 |
|  |  |  |  |  |  |
| **severe coding variants** | | | | | |
| **Gene** | **CaseOccurences** | **ControlOccurences** | **CaseTotal** | **ControlTotal** | **p_value** |
| SMAD4 | 3 | 0 | 113 | 300 | 0,0201 |
| PREX2 | 3 | 1 | 113 | 300 | 0,0642 |
| CACNA1D | 2 | 0 | 113 | 300 | 0,0744 |
| DNMT3A | 2 | 0 | 113 | 300 | 0,0744 |
| PRSS1 | 2 | 0 | 113 | 300 | 0,0744 |
| TP53 | 2 | 0 | 113 | 300 | 0,0744 |
|  |  |  |  |  |  |
| **regulatory variants** | | | | | |
| **Gene** | **CaseOccurences** | **ControlOccurences** | **CaseTotal** | **ControlTotal** | **p_value** |
| AKAP6 | 3 | 0 | 113 | 300 | 0,0201 |
| CCND3 | 3 | 0 | 113 | 300 | 0,0201 |
| SSBP2 | 3 | 0 | 113 | 300 | 0,0201 |
| ARID1A | 4 | 2 | 113 | 300 | 0,0502 |
| BCL3 | 3 | 1 | 113 | 300 | 0,0642 |
| ETV5 | 3 | 1 | 113 | 300 | 0,0642 |
| AXIN2 | 2 | 0 | 113 | 300 | 0,0744 |
| BCL11B | 2 | 0 | 113 | 300 | 0,0744 |
| HOXA13 | 2 | 0 | 113 | 300 | 0,0744 |
| SND1 | 2 | 0 | 113 | 300 | 0,0744 |
| SS18 | 2 | 0 | 113 | 300 | 0,0744 |

## Table S6: Validated genes in TCGA, having a p < 0.1 from the gene-wise burden tests from northern Sweden cohort, with either filtering criteria "coding variants" and "severe coding variants", and having a p < 0.1 in TCGA-gnomAD burden test, using either of the filtering criteria "coding variants", "severe coding variants

|  |  |  |  |  |  |  |
| --- | --- | --- | --- | --- | --- | --- |
| **Gene** | **CaseOccurences_TCGA_coding** | **ControlOccurences_gnomAD_coding** | **p_value_TCGA_coding** | **CaseOccurences_TCGA_severe** | **ControlOccurences_gnomAD_severe** | **p_value_TCGA_severe** |
| TP53 | 6 | 119 | 1,350E-02 | 4 | 47 | 8,033E-03 |
| CREBBP | 45 | 2090 | 3,497E-02 | 3 | 81 | 1,491E-01 |
| GAS7 | 15 | 298 | 1,497E-04 | 13 | 71 | 7,597E-10 |
| NBN | 31 | 1212 | 7,966E-03 | 5 | 132 | 6,325E-02 |
| STRN | 73 | 3579 | 4,374E-02 | 1 | 107 | 8,295E-01 |
|  |  |  |  |  |  |  |
|  |  |  |  |  |  |  |
| **Gene** | **CaseOccurences_TCGA_coding** | **ControlOccurences_gnomAD_coding** | **p_value_TCGA_coding** | **CaseOccurences_TCGA_severe** | **ControlOccurences_gnomAD_severe** | **p_value_TCGA_severe** |
| DNMT3A | 17 | 713 | 8,259E-02 | 10 | 229 | 5,355E-03 |
| TP53 | 6 | 119 | 1,350E-02 | 4 | 47 | 8,033E-03 |

## Table S7: Results from REGENIE using UK Biobank data, using any of the models “all”, “pLoF”, “pLoF + missense”.

| **CHROM** | **Gene** | **Variant_types_tested** | **BETA** | **SE** | **CHISQ** | **P** |
| --- | --- | --- | --- | --- | --- | --- |
| **17** | TP53 | pLoF+missense | 2,9400 | 1,0558 | 7,7542 | 0,0054 |
| **18** | SMAD4 | pLoF+missense | 1,0514 | 0,5660 | 3,4506 | 0,0632 |
| **18** | SMAD4 | all | 1,0514 | 0,5660 | 3,4506 | 0,0632 |
| **2** | DNMT3A | pLoF | 1,4978 | 0,8278 | 3,2738 | 0,0704 |
| **4** | PDGFRA | pLoF+missense | - 0,7110 | 0,4289 | 2,7485 | 0,0973 |
| **4** | PDGFRA | all | - 0,7110 | 0,4289 | 2,7485 | 0,0973 |
| **17** | RAD51C | pLoF+missense | - 0,6115 | 0,3829 | 2,5506 | 0,1103 |
| **17** | RAD51C | all | - 0,6115 | 0,3829 | 2,5506 | 0,1103 |
| **7** | CUX1 | pLoF+missense | 0,3457 | 0,2177 | 2,5226 | 0,1122 |
| **7** | CUX1 | all | 0,3306 | 0,2166 | 2,3288 | 0,1270 |
| **14** | DICER1 | pLoF+missense | - 0,5041 | 0,3384 | 2,2198 | 0,1363 |
| **14** | DICER1 | all | - 0,5041 | 0,3384 | 2,2198 | 0,1363 |
| **5** | SDHA | pLoF+missense | - 0,5858 | 0,4047 | 2,0950 | 0,1478 |
| **5** | SDHA | all | - 0,5858 | 0,4047 | 2,0950 | 0,1478 |
| **7** | PRSS1 | pLoF+missense | - 1,1742 | 0,8379 | 1,9640 | 0,1611 |
| **7** | PRSS1 | all | - 1,1742 | 0,8379 | 1,9640 | 0,1611 |
| **9** | JAK2 | pLoF+missense | - 0,3377 | 0,2647 | 1,6282 | 0,2019 |
| **9** | JAK2 | all | - 0,3377 | 0,2647 | 1,6282 | 0,2019 |
| **15** | SPRED1 | pLoF+missense | - 0,7932 | 0,6542 | 1,4704 | 0,2253 |
| **15** | SPRED1 | all | - 0,7932 | 0,6542 | 1,4704 | 0,2253 |
| **5** | SDHA | pLoF | - 1,2260 | 1,0485 | 1,3672 | 0,2423 |
| **8** | RECQL4 | pLoF+missense | - 0,2572 | 0,2233 | 1,3272 | 0,2493 |
| **8** | RECQL4 | all | - 0,2572 | 0,2233 | 1,3272 | 0,2493 |
| **17** | BRCA1 | pLoF+missense | 0,5695 | 0,5029 | 1,2827 | 0,2574 |
| **17** | BRCA1 | all | 0,5695 | 0,5029 | 1,2827 | 0,2574 |
| **22** | LZTR1 | pLoF+missense | 0,6765 | 0,6045 | 1,2525 | 0,2631 |
| **17** | TP53 | all | 0,7795 | 0,6986 | 1,2449 | 0,2645 |
| **2** | ALK | pLoF+missense | 0,3876 | 0,3488 | 1,2348 | 0,2665 |
| **2** | ALK | all | 0,3876 | 0,3488 | 1,2348 | 0,2665 |
| **16** | FANCA | all | - 0,3852 | 0,3475 | 1,2289 | 0,2676 |
| **11** | CBL | pLoF+missense | - 0,5701 | 0,5245 | 1,1816 | 0,2770 |
| **11** | CBL | all | - 0,5701 | 0,5245 | 1,1816 | 0,2770 |
| **3** | COL7A1 | pLoF+missense | 0,1455 | 0,1483 | 0,9620 | 0,3267 |
| **3** | COL7A1 | all | 0,1455 | 0,1483 | 0,9620 | 0,3267 |
| **11** | KMT2A | pLoF | 1,5783 | 1,6418 | 0,9241 | 0,3364 |
| **16** | FANCA | pLoF | - 1,2015 | 1,2605 | 0,9086 | 0,3405 |
| **2** | BARD1 | pLoF+missense | - 0,2386 | 0,2555 | 0,8722 | 0,3503 |
| **2** | BARD1 | all | - 0,2386 | 0,2555 | 0,8722 | 0,3503 |
| **16** | FANCA | pLoF+missense | - 0,3418 | 0,3871 | 0,7795 | 0,3773 |
| **13** | RB1 | pLoF+missense | - 0,3032 | 0,3550 | 0,7295 | 0,3930 |
| **19** | ERCC2 | pLoF+missense | - 0,3165 | 0,3814 | 0,6885 | 0,4067 |
| **19** | BRD4 | pLoF+missense | - 0,2589 | 0,3251 | 0,6339 | 0,4259 |
| **19** | BRD4 | all | - 0,2589 | 0,3251 | 0,6339 | 0,4259 |
| **9** | XPA | pLoF+missense | - 0,6060 | 0,8073 | 0,5635 | 0,4528 |
| **9** | XPA | all | - 0,6060 | 0,8073 | 0,5635 | 0,4528 |
| **2** | BARD1 | pLoF | - 1,1303 | 1,5288 | 0,5467 | 0,4597 |
| **2** | ABCB11 | pLoF+missense | - 0,2847 | 0,3917 | 0,5283 | 0,4673 |
| **2** | ABCB11 | all | - 0,2847 | 0,3917 | 0,5283 | 0,4673 |
| **3** | XPC | pLoF | 1,0686 | 1,5099 | 0,5009 | 0,4791 |
| **4** | FGFR3 | all | - 0,3239 | 0,4689 | 0,4771 | 0,4897 |
| **12** | CLIP1 | pLoF | - 1,1352 | 1,6918 | 0,4502 | 0,5022 |
| **18** | WDR7 | pLoF+missense | - 0,2162 | 0,3227 | 0,4487 | 0,5029 |
| **18** | WDR7 | all | - 0,2162 | 0,3227 | 0,4487 | 0,5029 |
| **11** | NUP98 | pLoF+missense | - 0,2004 | 0,3043 | 0,4336 | 0,5102 |
| **11** | NUP98 | all | - 0,2004 | 0,3043 | 0,4336 | 0,5102 |
| **11** | MYO7A | pLoF | 0,6434 | 0,9812 | 0,4300 | 0,5120 |
| **19** | CEBPA | pLoF+missense | - 0,3797 | 0,6486 | 0,3427 | 0,5583 |
| **19** | CEBPA | all | - 0,3797 | 0,6486 | 0,3427 | 0,5583 |
| **5** | AFF4 | pLoF+missense | 0,2099 | 0,3613 | 0,3374 | 0,5613 |
| **5** | AFF4 | all | 0,2099 | 0,3613 | 0,3374 | 0,5613 |
| **22** | CHEK2 | pLoF+missense | - 0,2447 | 0,4356 | 0,3156 | 0,5743 |
| **22** | CHEK2 | all | - 0,2447 | 0,4356 | 0,3156 | 0,5743 |
| **22** | LZTR1 | all | 0,1958 | 0,3513 | 0,3105 | 0,5773 |
| **13** | RB1 | all | - 0,1842 | 0,3344 | 0,3035 | 0,5817 |
| **3** | MITF | pLoF+missense | 0,1934 | 0,3583 | 0,2914 | 0,5893 |
| **3** | MITF | all | 0,1934 | 0,3583 | 0,2914 | 0,5893 |
| **19** | ERCC2 | pLoF | - 1,0561 | 1,9925 | 0,2809 | 0,5961 |
| **3** | CACNA1D | all | - 0,1793 | 0,3580 | 0,2508 | 0,6165 |
| **5** | NSD1 | pLoF+missense | - 0,1416 | 0,2880 | 0,2417 | 0,6230 |
| **5** | NSD1 | all | - 0,1416 | 0,2880 | 0,2417 | 0,6230 |
| **11** | KMT2A | pLoF+missense | - 0,1039 | 0,2215 | 0,2202 | 0,6389 |
| **11** | KMT2A | all | - 0,1039 | 0,2215 | 0,2202 | 0,6389 |
| **4** | FGFR3 | pLoF+missense | - 0,2318 | 0,4942 | 0,2200 | 0,6391 |
| **19** | ERCC2 | all | - 0,1072 | 0,2382 | 0,2025 | 0,6527 |
| **3** | XPC | pLoF+missense | 0,2136 | 0,4904 | 0,1897 | 0,6632 |
| **3** | XPC | all | 0,2136 | 0,4904 | 0,1897 | 0,6632 |
| **17** | GAS7 | pLoF+missense | - 0,1998 | 0,4895 | 0,1666 | 0,6832 |
| **17** | GAS7 | all | - 0,1998 | 0,4895 | 0,1666 | 0,6832 |
| **11** | MYO7A | pLoF+missense | 0,0653 | 0,1634 | 0,1597 | 0,6894 |
| **11** | MYO7A | all | 0,0653 | 0,1634 | 0,1597 | 0,6894 |
| **10** | CCDC6 | pLoF+missense | 0,3311 | 0,8594 | 0,1484 | 0,7000 |
| **10** | CCDC6 | all | 0,3311 | 0,8594 | 0,1484 | 0,7000 |
| **8** | PREX2 | pLoF+missense | - 0,0650 | 0,1920 | 0,1147 | 0,7348 |
| **8** | PREX2 | all | - 0,0650 | 0,1920 | 0,1147 | 0,7348 |
| **3** | COL7A1 | pLoF | 0,2714 | 0,8543 | 0,1009 | 0,7507 |
| **12** | ERBB3 | pLoF+missense | - 0,0918 | 0,2910 | 0,0994 | 0,7525 |
| **12** | ERBB3 | all | - 0,0918 | 0,2910 | 0,0994 | 0,7525 |
| **1** | MPL | pLoF+missense | 0,1549 | 0,5190 | 0,0890 | 0,7654 |
| **1** | MPL | all | 0,1549 | 0,5190 | 0,0890 | 0,7654 |
| **21** | RUNX1 | pLoF+missense | 0,3041 | 1,2135 | 0,0628 | 0,8021 |
| **21** | RUNX1 | all | 0,3041 | 1,2135 | 0,0628 | 0,8021 |
| **8** | RECQL4 | pLoF | - 0,2514 | 1,0159 | 0,0612 | 0,8046 |
| **8** | NBN | pLoF+missense | - 0,0890 | 0,3682 | 0,0584 | 0,8090 |
| **8** | NBN | all | - 0,0890 | 0,3682 | 0,0584 | 0,8090 |
| **10** | PRF1 | pLoF+missense | 0,1004 | 0,5617 | 0,0320 | 0,8581 |
| **10** | PRF1 | all | 0,1004 | 0,5617 | 0,0320 | 0,8581 |
| **22** | ZC3H7B | pLoF+missense | - 0,0729 | 0,4405 | 0,0274 | 0,8686 |
| **22** | ZC3H7B | all | - 0,0729 | 0,4405 | 0,0274 | 0,8686 |
| **16** | CREBBP | pLoF+missense | - 0,0263 | 0,2093 | 0,0158 | 0,9001 |
| **16** | CREBBP | all | - 0,0263 | 0,2093 | 0,0158 | 0,9001 |
| **2** | DNMT3A | pLoF+missense | 0,0280 | 0,3922 | 0,0051 | 0,9430 |
| **2** | DNMT3A | all | 0,0280 | 0,3922 | 0,0051 | 0,9430 |
| **3** | CACNA1D | pLoF+missense | - 0,0261 | 0,3849 | 0,0046 | 0,9460 |
| **6** | POU5F1 | pLoF+missense | 0,0438 | 0,7904 | 0,0031 | 0,9559 |
| **6** | POU5F1 | all | 0,0438 | 0,7904 | 0,0031 | 0,9559 |
| **2** | STRN | pLoF+missense | - 0,0300 | 0,5417 | 0,0031 | 0,9559 |
| **2** | STRN | all | - 0,0300 | 0,5417 | 0,0031 | 0,9559 |
| **7** | SND1 | pLoF+missense | 0,0074 | 0,3680 | 0,0004 | 0,9839 |
| **7** | SND1 | all | 0,0074 | 0,3680 | 0,0004 | 0,9839 |

## Table S8: Occurence of CREBBP (chr16:3850637:G>A, p.Pro153Leu) recurring missense variant

| **Cohort** | **Case alleles with variant** | **Total case Alleles** | **Control alleles with variant** | **Total control alleles** | **Allele frequency in cases** | **Allele frequency in controls** |
| --- | --- | --- | --- | --- | --- | --- |
| Northern Sweden + Uppsala | 2 | 436 | 1 (Swegen) | 600 | 4,46E-03 | 5,00E-04 |
| TCGA | 4 | 1612 | 43 (gnomAD 2.1.1) | 102546 | 2,40E-03 | 4,30E-04 |
| UK Biobank | 2 | 1666 | 8 (UKB controls) | 16660 | 1,20E-03 | 4,80E-04 |

## Table S9: Second hit somatic mutations. Coding germline and somatic variant in same gene in same individual

| **gene** | **germline_pos** | **somatic_pos** | **diagnosis** |
| --- | --- | --- | --- |
| **TERT** | chr5:1278769:T:C | chr5:1260533:G:A | glioblastoma (grade 4) |
| **RHBDF2** | chr17:76479213:G:A | chr17:76476884:C:T | glioblastoma (grade 4) |
| **SOS1** | chr2:39007117:G:T | chr2:39056704:T:C | glioblastoma (grade 4) |
| **SUFU** | chr10:102504168:C:T | chr10:102599540:G:GC | glioblastoma (grade 4) |
| **RB1** | chr13:48364952:C:T | chr13:48381321:GC:G | glioblastoma (grade 4) |
