# Supplementary material for: Rare germline variants contribute to glioma predisposition: Whole-genome analysis of a regional cohort of glioma patients
Source: Neurooncol Adv. 2026 Feb 12;8(1):vdag038. doi: 10.1093/noajnl/vdag038 (PMC13007284; doi:10.1093/noajnl/vdag038)
Supplement: vdag038_Supplementary_Data [file vdag038_supplementary_data.zip › Table_S1.docx]

# Table S1: All genes included in downstream analyses

| **Rahman et al 2014** | **Melin et al 2015** | **GWAS-glioma** | **Choi et al 2023** | **Cosmic Census v98** | **All Unique** |
| --- | --- | --- | --- | --- | --- |
| ABCB11 | NF1 | AKAP6 | HERC2 | MLLT11 | ABCB11 |
| ALK | SPRED1 | AKT3 | TRPC4AP | BAP1 | ALK |
| APC | NF2 | C2orf80 | KIAA1549 | APC | APC |
| ATM | SMARCB1 | CCDC26 | MYO7A | CCNE1 | ATM |
| AXIN2 | LZTR1 | CDKN2A | PIK3R4 | SRC | AXIN2 |
| BAP1 | SMARCA4 | CDKN2B | ZC3H7B | PRRX1 | BAP1 |
| BLM | PTCH1 | CDKN2B-AS1 | PCNXL4 | DCTN1 | BLM |
| BMPR1A | SUFU | CYP4F12 | THSD7A | CNOT3 | BMPR1A |
| BRCA1 | TSC1 | D2HGDH | PCYT1A | PPFIBP1 | BRCA1 |
| BRCA2 | TSC2 | EGFR | IP6K1 | TCL1A | BRCA2 |
| BRIP1 | VHL | ETFA | ADAMTS8 | BAX | BRIP1 |
| BUB1B | TP53 | GRIA4 | AXIN2 | GNAS | BUB1B |
| CBL | MLH1 | HEATR3 | CDK4 | CHEK2 | CBL |
| CDC73 | MSH2 | IDH1 | DDN | NSD2 | CDC73 |
| CDH1 | PMS2 | LMF1 | MSH2 | HOOK3 | CDH1 |
| CDK4 | MSH6 | LRIG1 | PC | NRAS | CDK4 |
| CDKN1B | POT1 | MAML2 | HP1BP3 | STAT6 | CDKN1B |
| CDKN2A |  | MDM4 | SESTD1 | FOXL2 | CDKN2A |
| CEBPA |  | MPG | STK11 | CDH1 | CEBPA |
| CHEK2 |  | MYC | TRPC4AP | HMGA2 | CHEK2 |
| COL7A1 |  | NR | WDR7 | BRD3 | COL7A1 |
| CYLD |  | OBFC1 | ATM | H3F3A | CYLD |
| DDB2 |  | PHLDB1 | CHEK2 | HOXC13 | DDB2 |
| DICER1 |  | POLR3B | DMBT1 | SMAD3 | DICER1 |
| DIS3L2 |  | RAB27A | POT1 | APOBEC3B | DIS3L2 |
| DKC1 |  | RAVER2 |  | USP8 | DKC1 |
| DOCK8 |  | RHBDF1 |  | ASXL1 | DOCK8 |
| EGFR |  | RTEL1 |  | TP53 | EGFR |
| ELANE |  | SLC16A8 |  | PRDM16 | ELANE |
| ERCC2 |  | SSBP2 |  | ARID1B | ERCC2 |
| ERCC3 |  | STK38L |  | XPA | ERCC3 |
| ERCC4 |  | TERC |  | MLF1 | ERCC4 |
| ERCC5 |  | TERT |  | IGK | ERCC5 |
| EXT1 |  | RTEL1 |  | NUTM2B | EXT1 |
| EXT2 |  | TP53 |  | EIF3E | EXT2 |
| FAH |  | VTI1A |  | RNF43 | FAH |
| FANCA |  | ZBTB16 |  | OLIG2 | FANCA |
| FANCC |  |  |  | PAX5 | FANCC |
| FANCG |  |  |  | PTK6 | FANCG |
| FH |  |  |  | PDGFRB | FH |
| FLCN |  |  |  | ZRSR2 | FLCN |
| GATA2 |  |  |  | HIP1 | GATA2 |
| GBA |  |  |  | SOX2 | GBA |
| GJB2 |  |  |  | AMER1 | GJB2 |
| GPC3 |  |  |  | PTEN | GPC3 |
| HFE |  |  |  | CLTCL1 | HFE |
| HMBS |  |  |  | BCL2 | HMBS |
| HRAS |  |  |  | AR | HRAS |
| ITK |  |  |  | EIF4A2 | ITK |
| KIT |  |  |  | PPP2R1A | KIT |
| MAX |  |  |  | FAS | MAX |
| MEN1 |  |  |  | NUTM1 | MEN1 |
| MET |  |  |  | PHF6 | MET |
| MLH1 |  |  |  | P2RY8 | MLH1 |
| MSH2 |  |  |  | SRSF2 | MSH2 |
| MSH6 |  |  |  | MAF | MSH6 |
| MTAP |  |  |  | PPP6C | MTAP |
| MUTYH |  |  |  | POLD1 | MUTYH |
| NBN |  |  |  | TERT | NBN |
| NF1 |  |  |  | CDKN2A | NF1 |
| NF2 |  |  |  | LATS2 | NF2 |
| PALB2 |  |  |  | MAP2K4 | PALB2 |
| PDGFRA |  |  |  | DDX5 | PDGFRA |
| PHOX2B |  |  |  | PBX1 | PHOX2B |
| PMS2 |  |  |  | ACKR3 | PMS2 |
| POLD1 |  |  |  | SETD2 | POLD1 |
| POLE |  |  |  | CDKN1B | POLE |
| POLH |  |  |  | TSC1 | POLH |
| PRKAR1A |  |  |  | ABL2 | PRKAR1A |
| PRSS1 |  |  |  | MLLT6 | PRSS1 |
| PTCH1 |  |  |  | CEBPA | PTCH1 |
| PTEN |  |  |  | FBXO11 | PTEN |
| PTPN11 |  |  |  | TOP1 | PTPN11 |
| RAD51C |  |  |  | FAT1 | RAD51C |
| RAD51D |  |  |  | TNFRSF14 | RAD51D |
| RB1 |  |  |  | PIK3CA | RB1 |
| RECQL4 |  |  |  | NTRK1 | RECQL4 |
| RET |  |  |  | MDM4 | RET |
| RHBDF2 |  |  |  | KNL1 | RHBDF2 |
| RMRP |  |  |  | EZR | RMRP |
| RUNX1 |  |  |  | NIN | RUNX1 |
| SBDS |  |  |  | TMEM127 | SBDS |
| SDHA |  |  |  | AKT2 | SDHA |
| SDHAF2 |  |  |  | DDB2 | SDHAF2 |
| SDHB |  |  |  | BCL3 | SDHB |
| SDHC |  |  |  | HOXA9 | SDHC |
| SDHD |  |  |  | HLF | SDHD |
| SERPINA1 |  |  |  | RBM15 | SERPINA1 |
| SH2D1A |  |  |  | TPM4 | SH2D1A |
| SLC25A13 |  |  |  | TPR | SLC25A13 |
| SMAD4 |  |  |  | TET2 | SMAD4 |
| SMARCA4 |  |  |  | SDHB | SMARCA4 |
| SMARCB1 |  |  |  | FUS | SMARCB1 |
| SMARCE1 |  |  |  | LIFR | SMARCE1 |
| SOS1 |  |  |  | FHIT | SOS1 |
| SRY |  |  |  | MSI2 | SRY |
| STAT3 |  |  |  | ERCC5 | STAT3 |
| STK11 |  |  |  | MSN | STK11 |
| SUFU |  |  |  | GOLGA5 | SUFU |
| TERT |  |  |  | NCOA4 | TERT |
| TGFBR1 |  |  |  | SETBP1 | TGFBR1 |
| TMEM127 |  |  |  | POT1 | TMEM127 |
| TNFRSF6 (FAS) |  |  |  | ZMYM2 | TNFRSF6 (FAS) |
| TP53 |  |  |  | BLM | TP53 |
| TRIM37 |  |  |  | ETV4 | TRIM37 |
| TSC1 |  |  |  | KMT2D | TSC1 |
| TSC2 |  |  |  | SSX1 | TSC2 |
| UROD |  |  |  | NBN | UROD |
| VHL |  |  |  | ETV6 | VHL |
| WAS |  |  |  | MET | WAS |
| WRN |  |  |  | DROSHA | WRN |
| WT1 |  |  |  | FANCA | WT1 |
| XPA |  |  |  | PATZ1 | XPA |
| XPC |  |  |  | MYH9 | XPC |
|  |  |  |  | FANCC | SPRED1 |
|  |  |  |  | NTRK3 | LZTR1 |
|  |  |  |  | MYCL | POT1 |
|  |  |  |  | TRIM33 | AKAP6 |
|  |  |  |  | CCNB1IP1 | AKT3 |
|  |  |  |  | PTPN13 | C2orf80 |
|  |  |  |  | WWTR1 | CCDC26 |
|  |  |  |  | DEK | CDKN2B |
|  |  |  |  | HMGA1 | CDKN2B-AS1 |
|  |  |  |  | PLCG1 | CYP4F12 |
|  |  |  |  | PCM1 | D2HGDH |
|  |  |  |  | PPM1D | ETFA |
|  |  |  |  | EXT2 | GRIA4 |
|  |  |  |  | TLX1 | HEATR3 |
|  |  |  |  | PAX3 | IDH1 |
|  |  |  |  | RAD51B | LMF1 |
|  |  |  |  | ROS1 | LRIG1 |
|  |  |  |  | TP63 | MAML2 |
|  |  |  |  | ACSL3 | MDM4 |
|  |  |  |  | SPOP | MPG |
|  |  |  |  | KDM6A | MYC |
|  |  |  |  | ATIC | NR |
|  |  |  |  | PDGFB | OBFC1 |
|  |  |  |  | KLF4 | PHLDB1 |
|  |  |  |  | SMO | POLR3B |
|  |  |  |  | FSTL3 | RAB27A |
|  |  |  |  | PBRM1 | RAVER2 |
|  |  |  |  | FGFR4 | RHBDF1 |
|  |  |  |  | TMPRSS2 | RTEL1 |
|  |  |  |  | CDC73 | SLC16A8 |
|  |  |  |  | NR4A3 | SSBP2 |
|  |  |  |  | BCL9L | STK38L |
|  |  |  |  | DDX6 | TERC |
|  |  |  |  | AKT1 | VTI1A |
|  |  |  |  | PTPRC | ZBTB16 |
|  |  |  |  | NAB2 | HERC2 |
|  |  |  |  | POU5F1 | TRPC4AP |
|  |  |  |  | EGFR | KIAA1549 |
|  |  |  |  | RB1 | MYO7A |
|  |  |  |  | KIT | PIK3R4 |
|  |  |  |  | HNF1A | ZC3H7B |
|  |  |  |  | TFG | PCNXL4 |
|  |  |  |  | MALT1 | THSD7A |
|  |  |  |  | MAPK1 | PCYT1A |
|  |  |  |  | FOXO1 | IP6K1 |
|  |  |  |  | SBDS | ADAMTS8 |
|  |  |  |  | RPN1 | DDN |
|  |  |  |  | ASPSCR1 | PC |
|  |  |  |  | RUNX1T1 | HP1BP3 |
|  |  |  |  | CRTC3 | SESTD1 |
|  |  |  |  | MRTFA | WDR7 |
|  |  |  |  | NOTCH2 | DMBT1 |
|  |  |  |  | TCF7L2 | MLLT11 |
|  |  |  |  | GPC3 | CCNE1 |
|  |  |  |  | ACVR1 | SRC |
|  |  |  |  | ERCC2 | PRRX1 |
|  |  |  |  | TCEA1 | DCTN1 |
|  |  |  |  | SLC34A2 | CNOT3 |
|  |  |  |  | ARHGEF12 | PPFIBP1 |
|  |  |  |  | HSP90AA1 | TCL1A |
|  |  |  |  | RPL10 | BAX |
|  |  |  |  | LMO1 | GNAS |
|  |  |  |  | LZTR1 | NSD2 |
|  |  |  |  | CDK6 | HOOK3 |
|  |  |  |  | ETV1 | NRAS |
|  |  |  |  | KLF6 | STAT6 |
|  |  |  |  | SET | FOXL2 |
|  |  |  |  | FCRL4 | HMGA2 |
|  |  |  |  | DDR2 | BRD3 |
|  |  |  |  | IL6ST | H3F3A |
|  |  |  |  | MSH2 | HOXC13 |
|  |  |  |  | NCOA1 | SMAD3 |
|  |  |  |  | CD79B | APOBEC3B |
|  |  |  |  | FOXO3 | USP8 |
|  |  |  |  | MAP2K1 | ASXL1 |
|  |  |  |  | IL7R | PRDM16 |
|  |  |  |  | KDR | ARID1B |
|  |  |  |  | SOCS1 | MLF1 |
|  |  |  |  | COL1A1 | IGK |
|  |  |  |  | AXIN2 | NUTM2B |
|  |  |  |  | PIM1 | EIF3E |
|  |  |  |  | STAT5B | RNF43 |
|  |  |  |  | FGFR1OP | OLIG2 |
|  |  |  |  | SDC4 | PAX5 |
|  |  |  |  | TCF3 | PTK6 |
|  |  |  |  | PAX8 | PDGFRB |
|  |  |  |  | GATA1 | ZRSR2 |
|  |  |  |  | MEN1 | HIP1 |
|  |  |  |  | IL2 | SOX2 |
|  |  |  |  | ATR | AMER1 |
|  |  |  |  | NONO | CLTCL1 |
|  |  |  |  | TRA | BCL2 |
|  |  |  |  | BUB1B | AR |
|  |  |  |  | RUNX1 | EIF4A2 |
|  |  |  |  | MAX | PPP2R1A |
|  |  |  |  | ABI1 | FAS |
|  |  |  |  | TNFRSF17 | NUTM1 |
|  |  |  |  | ERG | PHF6 |
|  |  |  |  | ARID1A | P2RY8 |
|  |  |  |  | ITK | SRSF2 |
|  |  |  |  | LMNA | MAF |
|  |  |  |  | MDM2 | PPP6C |
|  |  |  |  | NF2 | LATS2 |
|  |  |  |  | FH | MAP2K4 |
|  |  |  |  | FANCG | DDX5 |
|  |  |  |  | FUBP1 | PBX1 |
|  |  |  |  | QKI | ACKR3 |
|  |  |  |  | SUZ12 | SETD2 |
|  |  |  |  | GOPC | ABL2 |
|  |  |  |  | JUN | MLLT6 |
|  |  |  |  | FANCF | FBXO11 |
|  |  |  |  | NCOA2 | TOP1 |
|  |  |  |  | MSH6 | FAT1 |
|  |  |  |  | GRIN2A | TNFRSF14 |
|  |  |  |  | SND1 | PIK3CA |
|  |  |  |  | KLK2 | NTRK1 |
|  |  |  |  | CANT1 | KNL1 |
|  |  |  |  | DNAJB1 | EZR |
|  |  |  |  | CRLF2 | NIN |
|  |  |  |  | RNF213 | AKT2 |
|  |  |  |  | STRN | BCL3 |
|  |  |  |  | YWHAE | HOXA9 |
|  |  |  |  | RPL22 | HLF |
|  |  |  |  | FOXO4 | RBM15 |
|  |  |  |  | PALB2 | TPM4 |
|  |  |  |  | KAT6B | TPR |
|  |  |  |  | IRS4 | TET2 |
|  |  |  |  | HOXA13 | FUS |
|  |  |  |  | SDHA | LIFR |
|  |  |  |  | WT1 | FHIT |
|  |  |  |  | WAS | MSI2 |
|  |  |  |  | EWSR1 | MSN |
|  |  |  |  | UBR5 | GOLGA5 |
|  |  |  |  | RAD21 | NCOA4 |
|  |  |  |  | RPL5 | SETBP1 |
|  |  |  |  | PRKACA | ZMYM2 |
|  |  |  |  | IDH2 | ETV4 |
|  |  |  |  | FLT4 | KMT2D |
|  |  |  |  | ERC1 | SSX1 |
|  |  |  |  | FANCE | ETV6 |
|  |  |  |  | ELF4 | DROSHA |
|  |  |  |  | NUP98 | PATZ1 |
|  |  |  |  | MUC1 | MYH9 |
|  |  |  |  | TRB | NTRK3 |
|  |  |  |  | TRD | MYCL |
|  |  |  |  | HERPUD1 | TRIM33 |
|  |  |  |  | DNM2 | CCNB1IP1 |
|  |  |  |  | SMAD2 | PTPN13 |
|  |  |  |  | GPHN | WWTR1 |
|  |  |  |  | IKZF1 | DEK |
|  |  |  |  | TFE3 | HMGA1 |
|  |  |  |  | MYD88 | PLCG1 |
|  |  |  |  | MAFB | PCM1 |
|  |  |  |  | BARD1 | PPM1D |
|  |  |  |  | KDSR | TLX1 |
|  |  |  |  | MPL | PAX3 |
|  |  |  |  | BCL6 | RAD51B |
|  |  |  |  | NUTM2D | ROS1 |
|  |  |  |  | SS18 | TP63 |
|  |  |  |  | CBLC | ACSL3 |
|  |  |  |  | BRIP1 | SPOP |
|  |  |  |  | IDH1 | KDM6A |
|  |  |  |  | FGFR2 | ATIC |
|  |  |  |  | CCND3 | PDGFB |
|  |  |  |  | POU2AF1 | KLF4 |
|  |  |  |  | RARA | SMO |
|  |  |  |  | CCND2 | FSTL3 |
|  |  |  |  | DDX3X | PBRM1 |
|  |  |  |  | PTPRK | FGFR4 |
|  |  |  |  | LMO2 | TMPRSS2 |
|  |  |  |  | POLE | NR4A3 |
|  |  |  |  | RBM10 | BCL9L |
|  |  |  |  | KDM5C | DDX6 |
|  |  |  |  | MYO5A | AKT1 |
|  |  |  |  | JAK1 | PTPRC |
|  |  |  |  | FOXP1 | NAB2 |
|  |  |  |  | PIK3CB | POU5F1 |
|  |  |  |  | SPEN | HNF1A |
|  |  |  |  | CCDC6 | TFG |
|  |  |  |  | CYLD | MALT1 |
|  |  |  |  | TCF12 | MAPK1 |
|  |  |  |  | ZFHX3 | FOXO1 |
|  |  |  |  | FANCD2 | RPN1 |
|  |  |  |  | AXIN1 | ASPSCR1 |
|  |  |  |  | ARNT | RUNX1T1 |
|  |  |  |  | MYB | CRTC3 |
|  |  |  |  | TFEB | MRTFA |
|  |  |  |  | XPC | NOTCH2 |
|  |  |  |  | NFKB2 | TCF7L2 |
|  |  |  |  | FES | ACVR1 |
|  |  |  |  | PICALM | TCEA1 |
|  |  |  |  | CXCR4 | SLC34A2 |
|  |  |  |  | SSX2 | ARHGEF12 |
|  |  |  |  | HRAS | HSP90AA1 |
|  |  |  |  | DAXX | RPL10 |
|  |  |  |  | ALK | LMO1 |
|  |  |  |  | AFF1 | CDK6 |
|  |  |  |  | TAF15 | ETV1 |
|  |  |  |  | CDX2 | KLF6 |
|  |  |  |  | SLC45A3 | SET |
|  |  |  |  | SS18L1 | FCRL4 |
|  |  |  |  | SH2B3 | DDR2 |
|  |  |  |  | SALL4 | IL6ST |
|  |  |  |  | PSIP1 | NCOA1 |
|  |  |  |  | NCOR2 | CD79B |
|  |  |  |  | CREB3L2 | FOXO3 |
|  |  |  |  | FGFR1 | MAP2K1 |
|  |  |  |  | PDCD1LG2 | IL7R |
|  |  |  |  | WRN | KDR |
|  |  |  |  | PTCH1 | SOCS1 |
|  |  |  |  | CNTRL | COL1A1 |
|  |  |  |  | KMT2C | PIM1 |
|  |  |  |  | RAF1 | STAT5B |
|  |  |  |  | ATP1A1 | FGFR1OP |
|  |  |  |  | BCL11A | SDC4 |
|  |  |  |  | FLCN | TCF3 |
|  |  |  |  | AFF3 | PAX8 |
|  |  |  |  | PML | GATA1 |
|  |  |  |  | EPAS1 | IL2 |
|  |  |  |  | ZBTB16 | ATR |
|  |  |  |  | CHCHD7 | NONO |
|  |  |  |  | MED12 | TRA |
|  |  |  |  | GAS7 | ABI1 |
|  |  |  |  | NUP214 | TNFRSF17 |
|  |  |  |  | BCR | ERG |
|  |  |  |  | FLI1 | ARID1A |
|  |  |  |  | TENT5C | LMNA |
|  |  |  |  | MAP3K1 | MDM2 |
|  |  |  |  | NFATC2 | FUBP1 |
|  |  |  |  | RABEP1 | QKI |
|  |  |  |  | ELL | SUZ12 |
|  |  |  |  | CASP8 | GOPC |
|  |  |  |  | MYC | JUN |
|  |  |  |  | BIRC3 | FANCF |
|  |  |  |  | FGFR3 | NCOA2 |
|  |  |  |  | LASP1 | GRIN2A |
|  |  |  |  | NFKBIE | SND1 |
|  |  |  |  | RAP1GDS1 | KLK2 |
|  |  |  |  | BRD4 | CANT1 |
|  |  |  |  | MLLT3 | DNAJB1 |
|  |  |  |  | IGL | CRLF2 |
|  |  |  |  | BRAF | RNF213 |
|  |  |  |  | GATA3 | STRN |
|  |  |  |  | FCGR2B | YWHAE |
|  |  |  |  | RET | RPL22 |
|  |  |  |  | ARID2 | FOXO4 |
|  |  |  |  | FLT3 | KAT6B |
|  |  |  |  | SYK | IRS4 |
|  |  |  |  | IRF4 | HOXA13 |
|  |  |  |  | DICER1 | EWSR1 |
|  |  |  |  | NFIB | UBR5 |
|  |  |  |  | LPP | RAD21 |
|  |  |  |  | LATS1 | RPL5 |
|  |  |  |  | GATA2 | PRKACA |
|  |  |  |  | LRIG3 | IDH2 |
|  |  |  |  | MTCP1 | FLT4 |
|  |  |  |  | B2M | ERC1 |
|  |  |  |  | MYH11 | FANCE |
|  |  |  |  | PER1 | ELF4 |
|  |  |  |  | RAC1 | NUP98 |
|  |  |  |  | CARS | MUC1 |
|  |  |  |  | CARD11 | TRB |
|  |  |  |  | MECOM | TRD |
|  |  |  |  | CDK12 | HERPUD1 |
|  |  |  |  | FOXA1 | DNM2 |
|  |  |  |  | MYOD1 | SMAD2 |
|  |  |  |  | NOTCH1 | GPHN |
|  |  |  |  | HEY1 | IKZF1 |
|  |  |  |  | CREB1 | TFE3 |
|  |  |  |  | STAT3 | MYD88 |
|  |  |  |  | BCOR | MAFB |
|  |  |  |  | LYL1 | BARD1 |
|  |  |  |  | ABL1 | KDSR |
|  |  |  |  | NFE2L2 | MPL |
|  |  |  |  | IKZF3 | BCL6 |
|  |  |  |  | MTOR | NUTM2D |
|  |  |  |  | SFPQ | SS18 |
|  |  |  |  | PTPN11 | CBLC |
|  |  |  |  | SMAD4 | FGFR2 |
|  |  |  |  | ARHGAP35 | CCND3 |
|  |  |  |  | MLLT10 | POU2AF1 |
|  |  |  |  | ERBB4 | RARA |
|  |  |  |  | ERCC3 | CCND2 |
|  |  |  |  | REL | DDX3X |
|  |  |  |  | DDX10 | PTPRK |
|  |  |  |  | STAG2 | LMO2 |
|  |  |  |  | PIK3R1 | RBM10 |
|  |  |  |  | MAP2K2 | KDM5C |
|  |  |  |  | CALR | MYO5A |
|  |  |  |  | PREX2 | JAK1 |
|  |  |  |  | TBL1XR1 | FOXP1 |
|  |  |  |  | NF1 | PIK3CB |
|  |  |  |  | RSPO2 | SPEN |
|  |  |  |  | KAT6A | CCDC6 |
|  |  |  |  | MLH1 | TCF12 |
|  |  |  |  | PDE4DIP | ZFHX3 |
|  |  |  |  | HOXD11 | FANCD2 |
|  |  |  |  | PTPRT | AXIN1 |
|  |  |  |  | TAL1 | ARNT |
|  |  |  |  | HLA-A | MYB |
|  |  |  |  | ACVR2A | TFEB |
|  |  |  |  | AFDN | NFKB2 |
|  |  |  |  | SH3GL1 | FES |
|  |  |  |  | XPO1 | PICALM |
|  |  |  |  | CDKN2C | CXCR4 |
|  |  |  |  | SIX1 | SSX2 |
|  |  |  |  | EML4 | DAXX |
|  |  |  |  | ATM | AFF1 |
|  |  |  |  | TRIP11 | TAF15 |
|  |  |  |  | CLIP1 | CDX2 |
|  |  |  |  | ETV5 | SLC45A3 |
|  |  |  |  | CBFB | SS18L1 |
|  |  |  |  | TRIM24 | SH2B3 |
|  |  |  |  | SFRP4 | SALL4 |
|  |  |  |  | CDH11 | PSIP1 |
|  |  |  |  | CDK4 | NCOR2 |
|  |  |  |  | ATF1 | CREB3L2 |
|  |  |  |  | PPARG | FGFR1 |
|  |  |  |  | KIF5B | PDCD1LG2 |
|  |  |  |  | NDRG1 | CNTRL |
|  |  |  |  | BCL11B | KMT2C |
|  |  |  |  | STK11 | RAF1 |
|  |  |  |  | CUX1 | ATP1A1 |
|  |  |  |  | SDHD | BCL11A |
|  |  |  |  | IGH | AFF3 |
|  |  |  |  | SRSF3 | PML |
|  |  |  |  | TBX3 | EPAS1 |
|  |  |  |  | CBFA2T3 | CHCHD7 |
|  |  |  |  | CNBP | MED12 |
|  |  |  |  | LCK | GAS7 |
|  |  |  |  | CD79A | NUP214 |
|  |  |  |  | TPM3 | BCR |
|  |  |  |  | HSP90AB1 | FLI1 |
|  |  |  |  | JAK2 | TENT5C |
|  |  |  |  | ZNF521 | MAP3K1 |
|  |  |  |  | PHOX2B | NFATC2 |
|  |  |  |  | CREB3L1 | RABEP1 |
|  |  |  |  | CTCF | ELL |
|  |  |  |  | HIF1A | CASP8 |
|  |  |  |  | MUTYH | BIRC3 |
|  |  |  |  | SUFU | FGFR3 |
|  |  |  |  | CACNA1D | LASP1 |
|  |  |  |  | BCL10 | NFKBIE |
|  |  |  |  | MLLT1 | RAP1GDS1 |
|  |  |  |  | TGFBR2 | BRD4 |
|  |  |  |  | BCL9 | MLLT3 |
|  |  |  |  | TSC2 | IGL |
|  |  |  |  | CRTC1 | BRAF |
|  |  |  |  | MITF | GATA3 |
|  |  |  |  | BTG1 | FCGR2B |
|  |  |  |  | TSHR | ARID2 |
|  |  |  |  | ERBB3 | FLT3 |
|  |  |  |  | NPM1 | SYK |
|  |  |  |  | VHL | IRF4 |
|  |  |  |  | NTRK2 | NFIB |
|  |  |  |  | ZNF384 | LPP |
|  |  |  |  | JAK3 | LATS1 |
|  |  |  |  | ARHGAP26 | LRIG3 |
|  |  |  |  | RANBP2 | MTCP1 |
|  |  |  |  | CBL | B2M |
|  |  |  |  | KCNJ5 | MYH11 |
|  |  |  |  | IL21R | PER1 |
|  |  |  |  | RSPO3 | RAC1 |
|  |  |  |  | RHOA | CARS |
|  |  |  |  | CD274 | CARD11 |
|  |  |  |  | DDIT3 | MECOM |
|  |  |  |  | KTN1 | CDK12 |
|  |  |  |  | HNRNPA2B1 | FOXA1 |
|  |  |  |  | NSD3 | MYOD1 |
|  |  |  |  | ESR1 | NOTCH1 |
|  |  |  |  | STIL | HEY1 |
|  |  |  |  | SMARCA4 | CREB1 |
|  |  |  |  | PDGFRA | BCOR |
|  |  |  |  | CIC | LYL1 |
|  |  |  |  | SDHAF2 | ABL1 |
|  |  |  |  | ATP2B3 | NFE2L2 |
|  |  |  |  | TRRAP | IKZF3 |
|  |  |  |  | EPS15 | MTOR |
|  |  |  |  | CD74 | SFPQ |
|  |  |  |  | BCL7A | ARHGAP35 |
|  |  |  |  | U2AF1 | MLLT10 |
|  |  |  |  | PMS2 | ERBB4 |
|  |  |  |  | LRP1B | REL |
|  |  |  |  | ETNK1 | DDX10 |
|  |  |  |  | ZNF331 | STAG2 |
|  |  |  |  | SMARCB1 | PIK3R1 |
|  |  |  |  | RECQL4 | MAP2K2 |
|  |  |  |  | CSF3R | CALR |
|  |  |  |  | COL2A1 | PREX2 |
|  |  |  |  | IKBKB | TBL1XR1 |
|  |  |  |  | ERCC4 | RSPO2 |
|  |  |  |  | TRAF7 | KAT6A |
|  |  |  |  | WDCP | PDE4DIP |
|  |  |  |  | MAP3K13 | HOXD11 |
|  |  |  |  | CTNNB1 | PTPRT |
|  |  |  |  | HOXD13 | TAL1 |
|  |  |  |  | SMARCE1 | HLA-A |
|  |  |  |  | ELK4 | ACVR2A |
|  |  |  |  | CBLB | AFDN |
|  |  |  |  | KRAS | SH3GL1 |
|  |  |  |  | EXT1 | XPO1 |
|  |  |  |  | CREBBP | CDKN2C |
|  |  |  |  | NCOR1 | SIX1 |
|  |  |  |  | PRF1 | EML4 |
|  |  |  |  | BCORL1 | TRIP11 |
|  |  |  |  | NT5C2 | CLIP1 |
|  |  |  |  | PRKAR1A | ETV5 |
|  |  |  |  | ERBB2 | CBFB |
|  |  |  |  | CHD4 | TRIM24 |
|  |  |  |  | SSX4 | SFRP4 |
|  |  |  |  | LEF1 | CDH11 |
|  |  |  |  | NKX2-1 | ATF1 |
|  |  |  |  | MYCN | PPARG |
|  |  |  |  | BTK | KIF5B |
|  |  |  |  | PRDM1 | NDRG1 |
|  |  |  |  | USP6 | BCL11B |
|  |  |  |  | PAFAH1B2 | CUX1 |
|  |  |  |  | CCND1 | IGH |
|  |  |  |  | KMT2A | SRSF3 |
|  |  |  |  | SF3B1 | TBX3 |
|  |  |  |  | TAL2 | CBFA2T3 |
|  |  |  |  | GNA11 | CNBP |
|  |  |  |  | CIITA | LCK |
|  |  |  |  | BMPR1A | CD79A |
|  |  |  |  | MN1 | TPM3 |
|  |  |  |  | TNFAIP3 | HSP90AB1 |
|  |  |  |  | FIP1L1 | JAK2 |
|  |  |  |  | CAMTA1 | ZNF521 |
|  |  |  |  | NSD1 | CREB3L1 |
|  |  |  |  | PLAG1 | CTCF |
|  |  |  |  | FBXW7 | HIF1A |
|  |  |  |  | TRIM27 | CACNA1D |
|  |  |  |  | TLX3 | BCL10 |
|  |  |  |  | EP300 | MLLT1 |
|  |  |  |  | HIST1H3B | TGFBR2 |
|  |  |  |  | FEV | BCL9 |
|  |  |  |  | PAX7 | CRTC1 |
|  |  |  |  | WIF1 | MITF |
|  |  |  |  | PTPRB | BTG1 |
|  |  |  |  | CLTC | TSHR |
|  |  |  |  | NUMA1 | ERBB3 |
|  |  |  |  | HOXC11 | NPM1 |
|  |  |  |  | RMI2 | NTRK2 |
|  |  |  |  | RHOH | ZNF384 |
|  |  |  |  | BRCA1 | JAK3 |
|  |  |  |  | ATRX | ARHGAP26 |
|  |  |  |  | EZH2 | RANBP2 |
|  |  |  |  | KEAP1 | KCNJ5 |
|  |  |  |  | H3F3B | IL21R |
|  |  |  |  | SDHC | RSPO3 |
|  |  |  |  | POLQ | RHOA |
|  |  |  |  | MAML2 | CD274 |
|  |  |  |  | TET1 | DDIT3 |
|  |  |  |  | FAT4 | KTN1 |
|  |  |  |  | HOXA11 | HNRNPA2B1 |
|  |  |  |  | AFF4 | NSD3 |
|  |  |  |  | DNMT3A | ESR1 |
|  |  |  |  | KDM5A | STIL |
|  |  |  |  | EBF1 | CIC |
|  |  |  |  | NRG1 | ATP2B3 |
|  |  |  |  | GNAQ | TRRAP |
|  |  |  |  | HIST1H4I | EPS15 |
|  |  |  |  | ACVR1B | CD74 |
|  |  |  |  | BRCA2 | BCL7A |
|  |  |  |  | PRCC | U2AF1 |
|  |  |  |  |  | LRP1B |
|  |  |  |  |  | ETNK1 |
|  |  |  |  |  | ZNF331 |
|  |  |  |  |  | CSF3R |
|  |  |  |  |  | COL2A1 |
|  |  |  |  |  | IKBKB |
|  |  |  |  |  | TRAF7 |
|  |  |  |  |  | WDCP |
|  |  |  |  |  | MAP3K13 |
|  |  |  |  |  | CTNNB1 |
|  |  |  |  |  | HOXD13 |
|  |  |  |  |  | ELK4 |
|  |  |  |  |  | CBLB |
|  |  |  |  |  | KRAS |
|  |  |  |  |  | CREBBP |
|  |  |  |  |  | NCOR1 |
|  |  |  |  |  | PRF1 |
|  |  |  |  |  | BCORL1 |
|  |  |  |  |  | NT5C2 |
|  |  |  |  |  | ERBB2 |
|  |  |  |  |  | CHD4 |
|  |  |  |  |  | SSX4 |
|  |  |  |  |  | LEF1 |
|  |  |  |  |  | NKX2-1 |
|  |  |  |  |  | MYCN |
|  |  |  |  |  | BTK |
|  |  |  |  |  | PRDM1 |
|  |  |  |  |  | USP6 |
|  |  |  |  |  | PAFAH1B2 |
|  |  |  |  |  | CCND1 |
|  |  |  |  |  | KMT2A |
|  |  |  |  |  | SF3B1 |
|  |  |  |  |  | TAL2 |
|  |  |  |  |  | GNA11 |
|  |  |  |  |  | CIITA |
|  |  |  |  |  | MN1 |
|  |  |  |  |  | TNFAIP3 |
|  |  |  |  |  | FIP1L1 |
|  |  |  |  |  | CAMTA1 |
|  |  |  |  |  | NSD1 |
|  |  |  |  |  | PLAG1 |
|  |  |  |  |  | FBXW7 |
|  |  |  |  |  | TRIM27 |
|  |  |  |  |  | TLX3 |
|  |  |  |  |  | EP300 |
|  |  |  |  |  | HIST1H3B |
|  |  |  |  |  | FEV |
|  |  |  |  |  | PAX7 |
|  |  |  |  |  | WIF1 |
|  |  |  |  |  | PTPRB |
|  |  |  |  |  | CLTC |
|  |  |  |  |  | NUMA1 |
|  |  |  |  |  | HOXC11 |
|  |  |  |  |  | RMI2 |
|  |  |  |  |  | RHOH |
|  |  |  |  |  | ATRX |
|  |  |  |  |  | EZH2 |
|  |  |  |  |  | KEAP1 |
|  |  |  |  |  | H3F3B |
|  |  |  |  |  | POLQ |
|  |  |  |  |  | TET1 |
|  |  |  |  |  | FAT4 |
|  |  |  |  |  | HOXA11 |
|  |  |  |  |  | AFF4 |
|  |  |  |  |  | DNMT3A |
|  |  |  |  |  | KDM5A |
|  |  |  |  |  | EBF1 |
|  |  |  |  |  | NRG1 |
|  |  |  |  |  | GNAQ |
|  |  |  |  |  | HIST1H4I |
|  |  |  |  |  | ACVR1B |
|  |  |  |  |  | PRCC |
